# Prognosis Individualized: Survival predictions for WHO grade II and III gliomas with a machine learning-based web application

**DOI:** 10.1038/s41746-023-00948-y

**Published:** 2023-10-26

**Authors:** Mert Karabacak, Pemla Jagtiani, Alejandro Carrasquilla, Isabelle M. Germano, Konstantinos Margetis

**Affiliations:** 1 Department of Neurosurgery, Mount Sinai Health System, New York, 10029 NY USA; 2 School of Medicine, SUNY Downstate Health Sciences University, New York, 11203 NY USA

**Keywords:** Outcomes research, CNS cancer

## Abstract

WHO grade II and III gliomas demonstrate diverse biological behaviors resulting in variable survival outcomes. In the context of glioma prognosis, machine learning (ML) approaches could facilitate the navigation through the maze of factors influencing survival, aiding clinicians in generating more precise and personalized survival predictions. Here we report the utilization of ML models in predicting survival at 12, 24, 36, and 60 months following grade II and III glioma diagnosis. From the National Cancer Database, we analyze 10,001 WHO grade II and 11,456 grade III cranial gliomas. Using the area under the receiver operating characteristic (AUROC) values, we deploy the top-performing models in a web application for individualized predictions. SHapley Additive exPlanations (SHAP) enhance the interpretability of the models. Top-performing predictive models are the ones built with LightGBM and Random Forest algorithms. For grade II gliomas, the models yield AUROC values ranging from 0.813 to 0.896 for predicting mortality across different timeframes, and for grade III gliomas, the models yield AUROCs ranging from 0.855 to 0.878. ML models provide individualized survival forecasts for grade II and III glioma patients across multiple clinically relevant time points. The user-friendly web application represents a pioneering digital tool to potentially integrate predictive analytics into neuro-oncology clinical practice, to empower prognostication and personalize clinical decision-making.

## Introduction

The age-adjusted incidence of primary malignant central nervous system (CNS) tumors in the United States is approximately 7.1 per 100,000, according to recent statistics^1^. The vast majority of these primary CNS tumors are diffuse glioma^1^. Glioblastoma, classified as World Health Organization (WHO) grade IV, constitutes approximately 55% of gliomas, while the remaining 45% of glial tumors are composed of several various histologies, including grade II and grade III astrocytomas, oligodendrogliomas and oligoastrocytomas. These tumors encompass a diverse spectrum of infiltrative neoplasms exhibiting a myriad of biological characteristics and clinical behaviors, leading to a wide range of survival rates^2^. Due to this heterogeneity, there can be considerable uncertainty in prognostication and substantial challenges in assessing individual survival outcomes.

As we focus on prognostic modeling, machine learning (ML) techniques present several advantages over conventional statistical methods such as nomograms and regression-based models. Firstly, ML methods excel at processing large, complex, and heterogeneous datasets, detecting subtle patterns and associations that might be overlooked by conventional statistical methods^3,4^. Secondly, ML algorithms can incorporate a more extensive range of variables, including clinical, molecular, and imaging features, which could result in more comprehensive and individualized prognostic predictions^5–7^. Lastly, ML models can manage high-dimensional data without the strict assumptions required by traditional models. This means that ML is capable of identifying non-linear relationships and interactions between variables, providing more nuanced and intricate insights^3,4^.

In the context of glioma prognosis, ML approaches could facilitate the navigation through the maze of factors influencing survival, aiding clinicians in generating more precise and personalized survival predictions. As such, our objective is to leverage the power of ML algorithms to develop an accessible, user-friendly web application that aims to predict survival outcomes for patients with WHO grade II and III gliomas. By utilizing ML-based prediction models, we aim to address the challenges posed by the diversity and complexity inherent to the prognostication of gliomas and, ultimately, enhance patient care.

## Results

### Study Population

From the National Cancer Database (NCDB), a total of 10,001 histologically confirmed cranial WHO grade II gliomas and 11,456 histologically confirmed cranial WHO grade III gliomas were retrieved. The basic characteristics of the patient population before the application of the time point-specific exclusion criteria are presented in Table 1, while more detailed characteristics are presented in Supplementary Table 1. For patients with grade II glioma, the mean age was 42 ( ± 23), compared to a mean age of 51 ( ± 27) for those with grade III glioma. Among the grade II patients, 44.1% were female, while 44.5% of the grade III patients were female.

**Table 1:** Basic patient characteristics. For mortality outcomes, in cases where a patient was logged as alive, but their latest follow-up data [‘Last Contact or Death (Months from Diagnosis)’] was recorded prior to the specific survival time point in question, they were omitted from the pertinent survival analyses.

		Total (n = 21457)
Variables		Mean ( ± SD), Median (IQR), or n (%)
Age		47.0 ( ± 26.0)
Sex	Male	12002 (55.7%)
	Female	9555 (44.3%)
Histology	Anaplastic astrocytoma	8102 (37.6%)
	Diffuse astrocytoma	4291 (19.9%)
	Oligodendroglioma	4129 (19.2%)
	Anaplastic oligodendroglioma	2138 (9.9%)
	Oligoastrocytoma	1449 (6.7%)
	Anaplastic oligoastrocytoma	1216 (5.6%)
	Pleomorphic xanthoastrocytoma	232 (1.1%)
WHO Grade	Grade II	10001 (46.6%)
	Grade III	11456 (53.4%)
12-Month Mortality*	Yes	3496 (16.7%)
	No	17413 (83.3%)
24-Month Mortality*	Yes	5695 (27.9%)
	No	14710 (72.1%)
36-Month Mortality*	Yes	6984 (35.9%)
	No	12524 (64.1%)
60-Month Mortality*	Yes	8366 (52.8%)
	No	7492 (57.2%)

The numbers of included patients for each mortality outcome were delineated as follows: 9748 for 12-month mortality in grade II patients; 11161 for 12-month mortality in grade III patients; 9462 for 24-month mortality in grade II patients; 10943 for 24-month mortality in grade III patients; 8938 for 36-month mortality in grade II patients; 10572 for 36-month mortality in grade III patients; 6763 for 60-month mortality in grade II patients; and 9095 for 60-month mortality in grade III patients (SD, standard deviation; IQR, interquartile range n, number).

### Model Performances

The performance evaluation revealed that the top-performing models for each outcome were constructed using the LightGBM and Random Forest algorithms. For grade II gliomas, the Random Forest models yielded area under the receiver operating characteristics (AUROCs) of 0.888 [95% confidence interval (CI), 0.856–0.912] and (95% CI, 0.815–0.863) for predicting 12- and 60-month mortality, respectively; and the LightGBM models yielded AUROCs of 0.859 (95% CI, 0.804–0.867) and 0.813 (95% CI, 0.777–0.835) for predicting 24- and 36-month mortality, respectively. In the case of grade III gliomas, the LightGBM models resulted in AUROCs of 0.876 (95% CI, 0.857–0.899) and 0.860 (95% CI, 0.834–0.87) for predicting 12- and 60-month mortality, respectively; and the Random Forest models yielded AUROCs of 0.855 (95% CI, 0.839–0.870) and 0.878 (0.857–0.885) for predicting 24- and 36-month mortality, respectively. These results demonstrate good discriminatory ability (AUROC > 0.8) in distinguishing patients who died within all of the intervals investigated in our study from those who did not. Detailed information on the performance metrics of the top-performing models for each mortality outcome is presented in Table 2, and the performance metrics of all the models are found in Supplementary Table 2. Supplementary Fig. 1 illustrates confusion matrices for the top-performing models, while Supplementary Figs. 2 through 9 display confusion matrices for the models constructed with other algorithms.

**Table 2:** Performance metrics of the models (CI, confidence interval; AUPRC, area under the precision-recall curve; AUROC, area under the receiver operating characteristics curve).

Grade	Outcome	Algorithm	Sensitivity (95% CI)	Specificity (95% CI)	Accuracy (95% CI)	AUPRC (95% CI)	AUROC (95% CI)	Brier Score (95% CI)
Grade II	12-Month Mortality	Random Forest	0.838 (0.822–0.854)	0.814 (0.797–0.831)	0.816 (0.799–0.833)	0.383 (0.361–0.405)	0.888 (0.856–0.912)	0.054 (0.044–0.064)
Grade II	24-Month Mortality	LightGBM	0.712 (0.692–0.732)	0.839 (0.822–0.856)	0.823 (0.806–0.84)	0.523 (0.5–0.546)	0.859 (0.804–0.867)	0.083 (0.071–0.095)
Grade II	36-Month Mortality	LightGBM	0.653 (0.631–0.675)	0.836 (0.819–0.853)	0.803 (0.785–0.821)	0.564 (0.541–0.587)	0.813 (0.777–0.835)	0.111 (0.096–0.126)
Grade II	60-Month Mortality	Random Forest	0.684 (0.659–0.709)	0.835 (0.815–0.855)	0.787 (0.765–0.809)	0.748 (0.725–0.771)	0.846 (0.815–0.863)	0.142 (0.123–0.161)
Grade III	12-Month Mortality	LightGBM	0.768 (0.75–0.786)	0.811 (0.795–0.827)	0.8 (0.783–0.817)	0.725 (0.706–0.744)	0.876 (0.857–0.889)	0.119 (0.106–0.132)
Grade III	24-Month Mortality	Random Forest	0.722 (0.703–0.741)	0.81 (0.794–0.826)	0.796 (0.779–0.813)	0.775 (0.758–0.792)	0.855 (0.839–0.87)	0.153 (0.138–0.168)
Grade III	36-Month Mortality	Random Forest	0.763 (0.745–0.781)	0.827 (0.811–0.843)	0.874 (0.86–0.888)	0.794 (0.777–0.811)	0.878 (0.857–0.885)	0.146 (0.131–0.161)
Grade III	60-Month Mortality	LightGBM	0.816 (0.798–0.834)	0.748 (0.728–0.768)	0.93 (0.918–0.942)	0.795 (0.776–0.814)	0.86 (0.834–0.87)	0.142 (0.126–0.158)

**Fig. 1:**
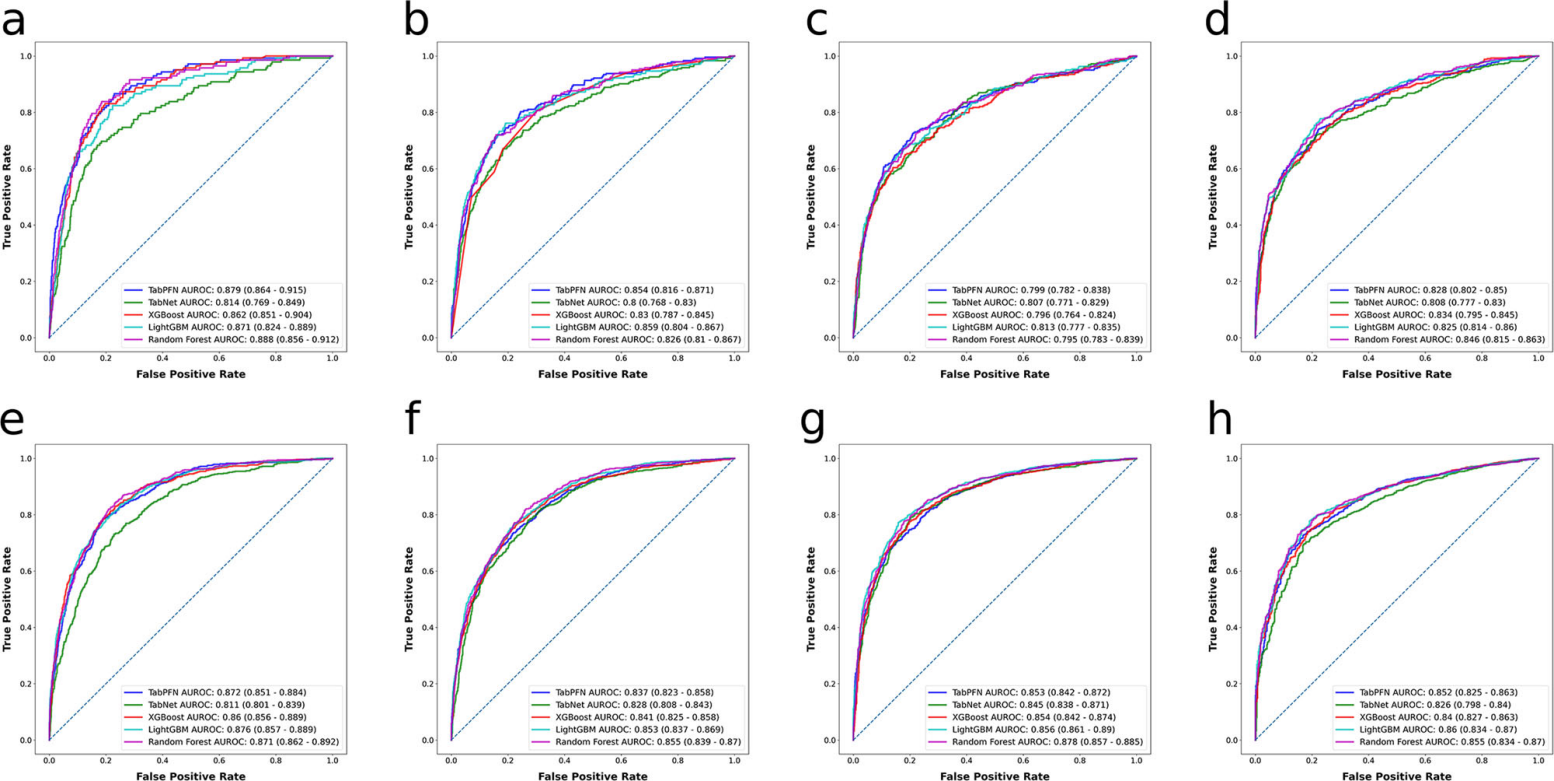
Algorithms’ receiver operating characteristics for predicting mortality at different time points for patients with WHO grade II and III gliomas. Shown are the receiver operating characteristic curves of the models built with different algorithms for **a** 12-month, **b** 24-month, **c** 36-month, and **d** 60-month mortality for patients with WHO grade II gliomas and for **e** 12-month, **f** 24-month, **g** 36-month, and **h** 60-month mortality for patients with WHO grade III gliomas. AUROC area under the receiver operating characteristic curve.

**Fig. 2:**
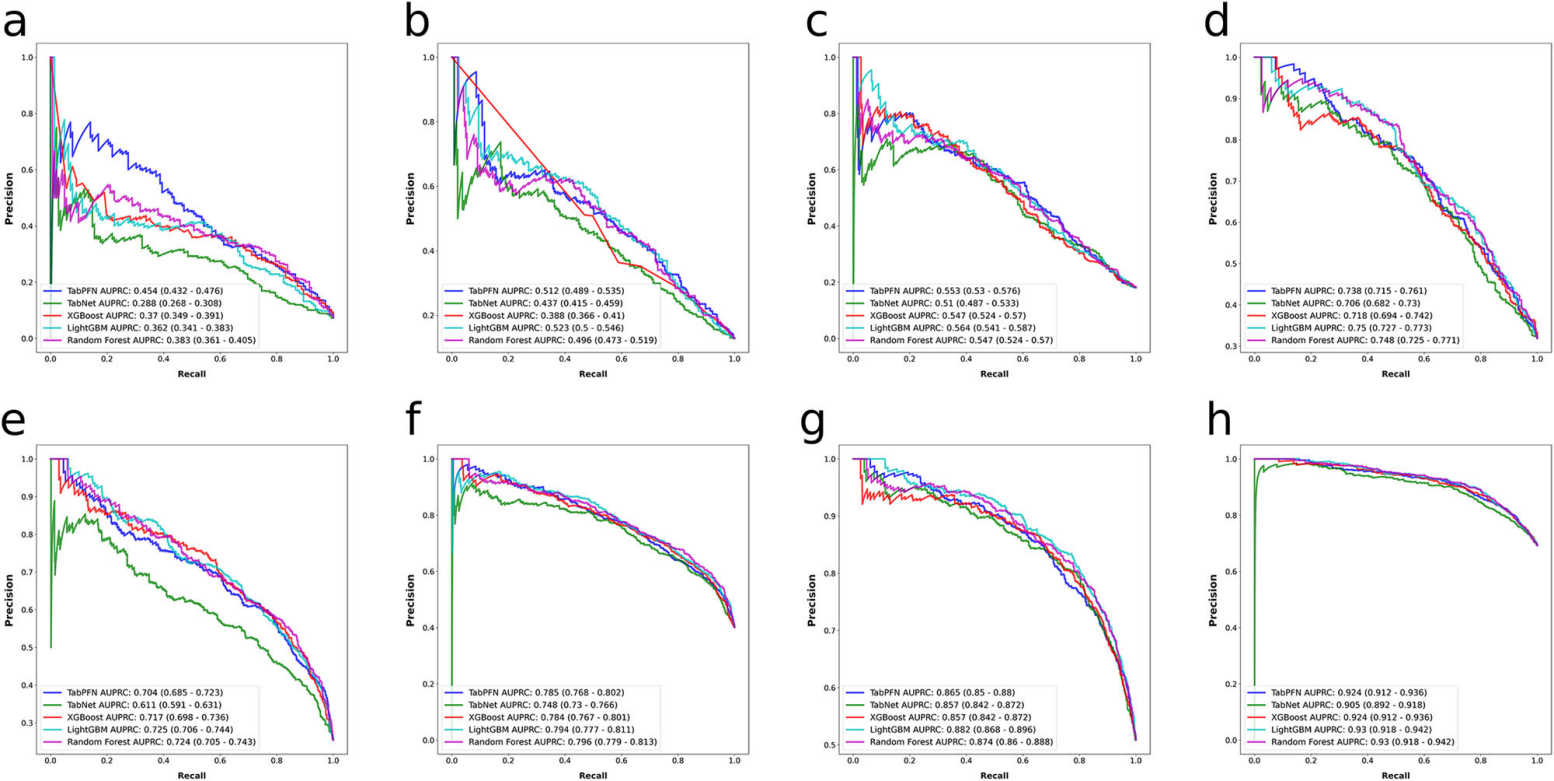
Algorithms’ precision–recall curves for predicting mortality at different time points for patients with WHO grade II and III gliomas. Shown are precision–recall curves of the models built with different algorithms for **a** 12-month, **b** 24-month, **c** 36-month, and **d** 60-month mortality for patients with WHO grade II gliomas and for **e** 12-month, **f** 24-month, **g** 36-month, and **h** 60-month mortality for patients with WHO grade III gliomas. AUPRC area under the precision–recall curve.

**Fig. 3:**
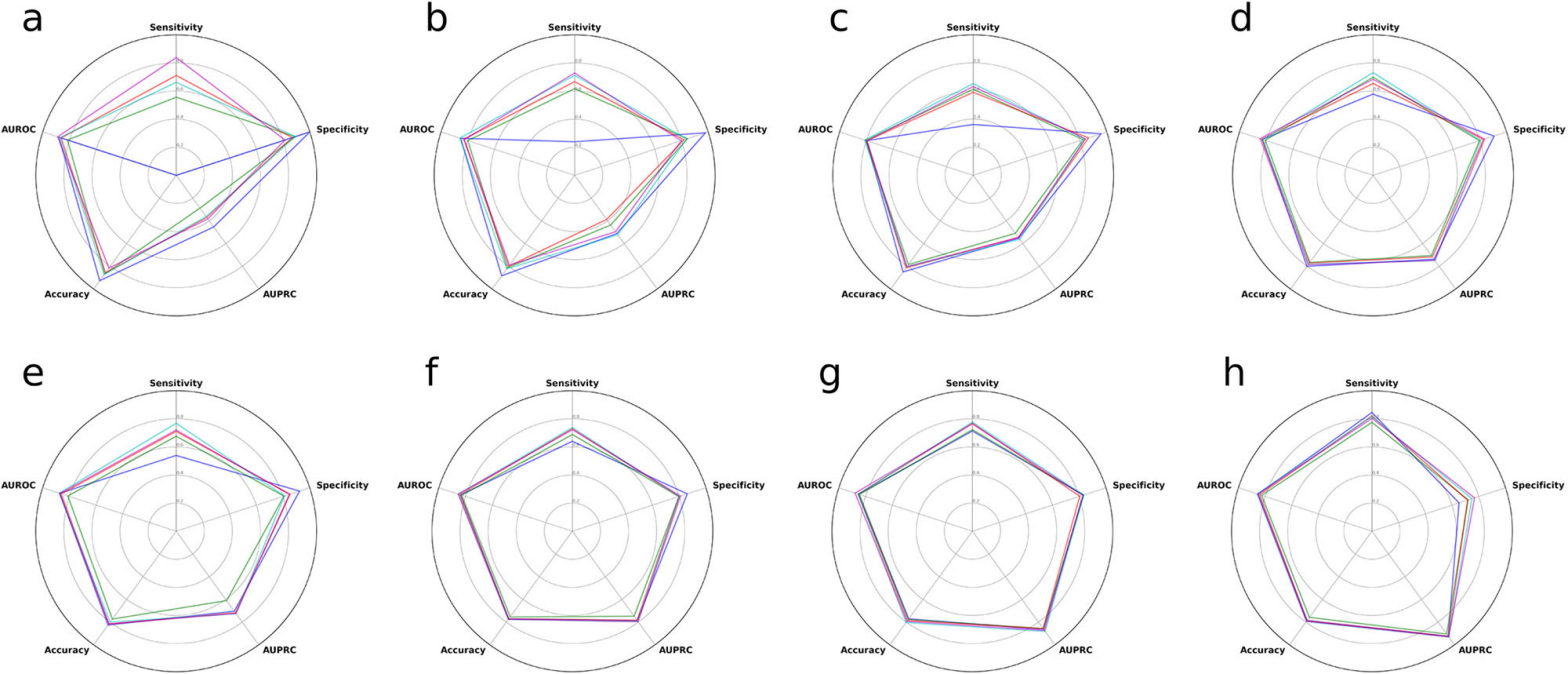
Radar plots showing the prediction performance of the models built with different algorithms across multiple metrics for predicting mortality at different time points for patients with WHO grade II and III gliomas. Shown are the radar plots of the models built with different algorithms for **a** 12-month, **b** 24-month, **c** 36-month, and **d** 60-month mortality for patients with WHO grade II gliomas and for **e** 12-month, **f** 24-month, **g** 36-month, and **h** 60-month mortality for patients with WHO grade III gliomas. AUROC area under the receiver operating characteristic curve, AUPRC area under the precision–recall curve.

Depicted in Figs. 1, 2 are the receiver operating characteristics (ROCs) and precision-recall curves (PRCs) corresponding to the quartet of survival outcomes for both grade II and grade III tumors, respectively. Figure 3 presents multiple radar charts, each corresponding to one of the four mortality outcomes of interest for both grade II and III tumors. These charts serve as an instrument for multidimensional visualization, with each of the five axes standing for a separate performance indicator. The placement on each respective axis signifies the model’s performance in relation to that particular indicator. Consequently, these radar charts enable a comparative analysis of model performance across various metrics.

**Fig. 4:**
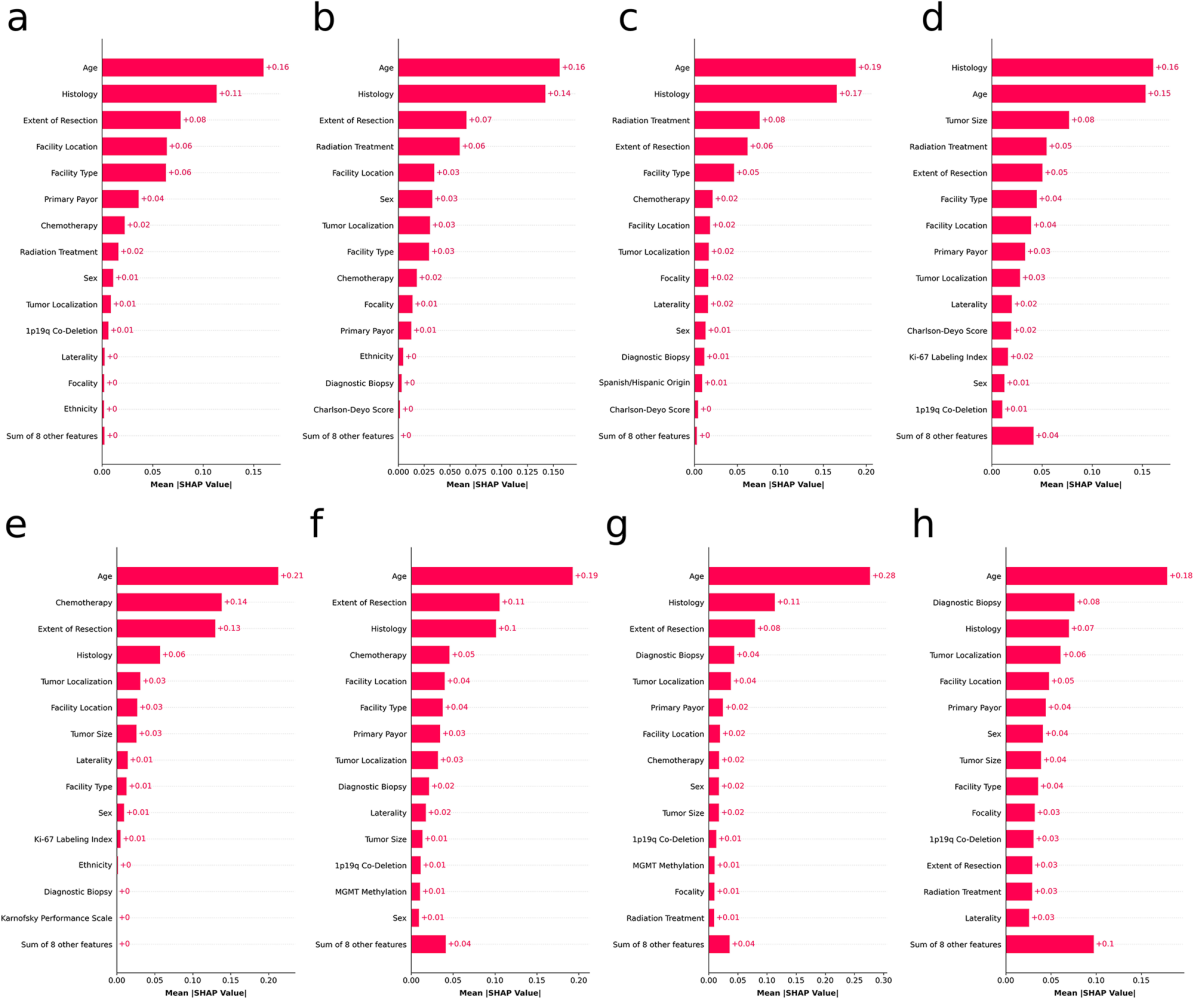
The 15 most important features and their mean SHAP values of the models built with different algorithms for predicting mortality at different time points for patients with WHO grade II and III gliomas. Shown are the top features for **a** Random Forest model predicting 12-month mortality, **b** LightGBM model predicting 24-month mortality, **c** LightGBM model predicting 36-month mortality, **d** Random Forest model predicting 60-month mortality for patients with WHO grade II gliomas; and for **e** LightGBM model predicting 12-month mortality, **f** Random Forest model predicting 24-month mortality, **g** Random Forest model predicting 36-month mortality, and **h** LightGBM model predicting 60-month mortality for patients with WHO grade III gliomas. SHAP SHapley Additive exPlanations.

Figure 4 illustrates SHapley Additive exPlanations (SHAP) bar plots for the top-performing models, which elucidate the aggregate influence of individual features on the predictions for each survival outcome. The length of each bar represents the mean SHAP value, signifying the intensity of a feature’s effect on the predicted outcome, with features arranged in order of their importance, the most critical being at the top. Supplementary Figs. 10–17 provide SHAP bar plots for all other algorithms pertaining to each outcome.

Partial dependence plots (PDPs) depict the isolated effect of a single feature on an ML model’s predicted outcome. The PDPs for the top-performing models can be found in Supplementary Figs. 18–25. These plots are crucial for interpreting the influence of individual features on the model’s predictions, revealing the correlation between a particular feature and the predicted outcome while concurrently nullifying the effects of other features.

## Discussion

This study introduces a suite of ML models adept at predicting survival prognoses for WHO grade II and III gliomas at 12, 24, 36, and 60 months subsequent to diagnosis. To ensure seamless integration into clinical environments, we devised a web application potentially enabling clinicians to individualize patient care and anticipate survival probabilities at specified intervals. This digital tool potentially streamlines collaborative decision-making processes with patients, ensuring they are well-informed regarding prospective survival outcomes post-diagnosis. These predictive indicators potentially furnish clinicians with the enhanced capacity to customize their care strategies, thereby optimizing the management of risk factors in high-risk patients.

A substantial body of literature exists on the topic of survival prediction in gliomas using various modalities. For instance, Zhao et al. utilized Cox Proportional Hazards, Support Vector Machine, and Random Forest algorithms on an extensive glioma dataset comprising 3462 patients^8^. Their objective was to determine the most efficacious method for survival prediction. Their models integrated widely-accepted variables commonly found in extensive brain tumor registries, such as age, sex, chemotherapy status, surgical resection status, radiation therapy status, tumor histology, and tumor location. Despite their assertion that the models’ predictive capacity was based solely on non-imaging and non-molecular data—data typically accessible in high-capacity cancer centers treating gliomas—the translatability of their findings remains ambiguous. This uncertainty arises because a clear means for clinicians to derive survival predictions—without a practical framework or the models’ source code—is absent. With that being said, the authors reported a survival prediction accuracy represented by a c-index (analogous to AUROC) that varied between 0.757 and 0.771, which our models outperformed by a considerable margin. Conversely, Gittleman et al. presented a nomogram, which could indeed help clinicians in real-world settings^9^. This tool employs clinicopathologic variables to calculate survival probabilities for WHO grade II and grade III glioma patients and even provides an online calculator similar to ours. Yet, the scope of their research is restricted by its limited generalizability due to the small sample size: 238 patients sourced from The Cancer Genome Atlas and the Ohio Brain Tumor Study. In comparison, our analyses drew from a more extensive dataset extracted from the NCDB, a database inclusive of data from over 1500 Commission on Cancer (CoC) accredited institutions.

Several different approaches have been proposed to predict survival for glioma patients by leveraging modalities beyond clinicopathologic variables, such as imaging and genomic data. Li et al. aimed to develop a radiomics-based model with preoperative T2-weighted MRIs of glioma patients, to prognosticate overall survival^10^. While this study did present a radiomics model with the distinct advantage of relying solely on preoperative data, it did not sufficiently elucidate how this model might be seamlessly integrated into clinical practice to inform prognosis and guide decision-making. Conversely, Xu et al. introduced an integrated methodology, amalgamating radiomics with clinical variables to establish a prognostic nomogram^11^. This nomogram is accessible for clinical application; however, it requires an input termed ‘Deep-radiomics Signature’. Even though Xu et al. asserted the reproducibility of their methodology in clinical practice, the means by which an individual clinician might procure this ‘Deep-radiomics Signature’ for nomogram use remains ambiguous. The literature offering predictive models based on genomic information is even broader, with numerous studies suggesting various gene signatures as predictive markers for survival outcomes in glioma patients^12–16^. While these gene signatures may offer valuable insights into the molecular underpinnings of the disease, their applicability is often restricted. This limitation arises because many of the genomic profiling techniques employed to formulate these signatures are infrequently incorporated into the standard care regimen for glioma patients. Consequently, these gene signature methodologies are predominantly relegated to the realm of feasibility studies and are seldom adopted in real-world clinical scenarios.

When it comes to interpreting the algorithm performances, Our findings that Random Forest and LightGBM models achieved the best performance aligns with previous studies showing the strengths of tree-based ensemble methods for clinical prediction tasks^17–19^. Both Random Forest and LightGBM build a large number of decision trees and leverage bagging and boosting techniques to improve prediction accuracy and limit overfitting^20,21^. The use of bagging in Random Forests, where each tree is trained on a random subset of features and samples, allows for effectively capturing non-linear relationships and complex interactions between predictors^21^. LightGBM enhances this further through gradient-based boosting, which selectively focuses on training examples with larger errors to minimize loss^20^. For Grade II tumors, Random Forest performed better for short-term (12-month) and long-term (60-month) mortality, while LightGBM was superior for medium-term (24- and 36-month) predictions. In contrast, for Grade III tumors, LightGBM was optimal for short and long-term predictions, while Random Forest excelled at medium-term forecasts. These differences may relate to variances in the complexity and progression patterns of low- versus high-grade gliomas^22^, causing each algorithm to have relative advantages at different prognostic time points. Overall, the high performance achieved by both approaches underscores the value of ensemble tree-based methods for risk stratification in glioma using demographic, clinical, and genomic variables.

The ML models, along with the corresponding web application, we introduce with this study, provide quantitative, personalized survival probability projections for WHO grade II and III glioma patients. This signifies a substantial leap beyond the traditional practice of stating generalized risks derived from studies averaging across heterogeneous populations. Another common practice often involves communicating risks qualitatively, perhaps supplemented by a quantitative assessment based on the clinician’s professional experience. However, depending exclusively on personal experience is limited by the inherently restricted patient population and potential biases of subjective risk appraisal. The predictions rendered by our models can be used to ascertain a patient’s prognosis at various intervals following their diagnosis, thereby enriching patient care. These predictive models can assist clinicians in pinpointing patients’ risk of poor survival outcomes, prioritizing their treatment, and strategizing their care pathways. For instance, these models can forewarn potential survival probabilities at specified time intervals, informing decisions about future care plans. Moreover, patients or their families at higher risk for poor survival outcomes can be offered intensive informed consent within a shared decision-making context. Beyond its potential application in shared decision-making processes, this approach presents opportunities for use in quality assurance. For instance, a pattern of poor survival outcomes in low-risk patients could have quality assurance implications, thereby prompting a reevaluation of care strategies. Consequently, our models could support the formulation of policies and procedures aimed at enhancing survival outcomes for low-risk patients, optimizing resource usage, and improving prognosis.

Our approach offers not only a precise and clinically viable methodology for predicting the survival outcomes for glioma patients but also enhances the interpretability of the predictions. The SHAP bar plots (Supplementary Figs. 10–17) provide comprehensive global explanations. In the realm of ML and model interpretations, global explanations aim to provide an overarching understanding of a model’s behavior across all its inputs. Rather than focusing on individual predictions, global explanations assess the entire dataset, revealing general patterns, tendencies, and relationships the model has learned. Conversely, the SHAP plots integrated into our web application deliver detailed local explanations, a feature that sets our approach apart. This enables users to gain specific insights into how individual predictions are influenced by distinct variables, allowing for a personalized, fine-grained understanding that has not been commonly available in previous models or applications. The integration of SHAP plots not only offers an additional layer of interpretability but also augments the trustworthiness of our model, especially when combined with clinical judgment. By assessing the predictor variables through SHAP, clinicians can confirm or challenge the model’s outputs based on their expertise, bridging the gap between machine-generated insights and human intuition. This synergy between SHAP and clinical assessment can significantly improve the model’s acceptance and reliance in real-world scenarios, underscoring its potential to assist clinicians in their decision-making processes.

In the global SHAP analyses of each mortality outcome, age emerged as the paramount predictor variable across all outcomes, except one, predicted by the top-performing models. This observation aligns with previous research that identified age as a salient prognostic indicator. For instance, Capelle et al. ascertained that individuals aged 55 and older presented an independent predictor of an adverse prognosis at the time of radiological diagnosis for WHO Grade II gliomas^23^. Similarly, Corell et al. discovered that the overall survival rate declined significantly for grade II glioma patients aged 60 and above, noting that negative preoperative prognostic factors (e.g., functional status and neurological deficit) become more prevalent with advancing age^24^. Histology emerged as another crucial predictor variable for both grade II and grade III tumors. According to the 2012–2016 edition of the Central Brain Tumor Registry of the United States (CBTRUS) Statistical Report, the 1-, 2-, and 5-year survival rates for diffuse astrocytomas stood at 74.7%, 64.1%, and 51.6%, respectively^25^. In contrast, the rates for oligodendrogliomas were 94.5%, 60.6%, and 82.7%. Regarding the grade III variants of these histologies, anaplastic astrocytomas demonstrated 1-, 2-, and 5-year survival rates of 64.3%, 46%, and 30.2%, respectively, whereas the rates for anaplastic oligodendrogliomas were 85.8%, 74.3%, and 60.2%. In a study conducted by Guo et al., results from their institutional cohort indicated that the median overall survival duration for grade II astrocytomas was 55.4 months, compared to 56.5 months for grade II oligodendrogliomas^26^. This difference did not exhibit statistical significance (*p* = 0.088). For grade III astrocytomas, the median survival duration was recorded as 53.6 months, while for grade III oligodendrogliomas, it was 45.8 months, again without reaching statistical significance. We found that the extent of resection was another influential variable among all outcomes. Jakola et al. conducted a study wherein grade II glioma patients were categorized into two cohorts: one underwent a biopsy with a subsequent wait-scan approach, while the other was subjected to an early resection^27^. The latter group exhibited a marked improvement in overall survival by an average of 7.1 years. Another study, after accounting for variables such as age, tumor location, and tumor subtype, affirmed that the extent of resection still holds significant sway over overall survival rates for grade II gliomas^28^. Parallel results concerning each of the predictor variables that were deemed to be important according to the SHAP analyses are documented extensively in the literature, elaborating on the specific elements influencing survival outcomes for both grade II and III gliomas. However, there exist contradictions within the literature. For instance, Jin et al. revealed a discernible uptick in both all-cause and tumor-specific mortality with age across seven age groups in their study scrutinizing risk factors for oligodendrogliomas across various age cohorts utilizing the Surveillance, Epidemiology, and End Results (SEER) database^29^. Contrarily, Jia et al., leveraging the same SEER database, ascertained a non-linear relationship between age and glioma prognosis^30^. Such disparate conclusions, even when sourced from an identical database like SEER, underscore a salient advantage of ML techniques: the capability to discern intricate, non-linear associations in datasets. Such complexities often prove more arduous to express and interpret via traditional methods like logistic regression.

The interpretations and implications of this study must be considered in light of certain limitations, predominantly associated with the inherent shortcomings and biases of retrospective database analyses^31–33^. The absence of molecular markers, such as the IDH molecular status, as well as the lack of clinical data, including imaging and information regarding the method used to determine the degree of tumor resection, and other patient performance status factors that could potentially influence the performance of the model, are notable limitations. The IDH mutation has been started to be collected from the year 2018, thus we could not include that as a variable since our analysis spanned 2010 to 2017^34^. The span of our analysis could have been increased to include the diagnoses after 2017; however, that would create a systematic bias in the missing data, which would impact the generalizability and robustness of the prediction models. As the amount and quality of data collected by national or international cancer registries continue to grow, future studies should take more predictive variables that could have clinical or molecular implications. The survival outcome data is confined to overall survival, thereby barring the analysis of WHO grade II and III gliomas’ progression-free survival or malignant transformation rates. Moreover, information regarding tumor management is limited to initial treatment regimens, rendering the impact of subsequent treatments unaccounted for. Pertinent details such as symptomatology, incidental presentation, involvement of eloquent structures, surgical considerations, and methods of the extent of resection (EOR) determination are conspicuously absent in the NCDB. While the NCDB collects data for roughly 70% of all new cancer diagnoses, it exclusively includes hospitals accredited by the CoC, which encompasses only about 30% of the approximately 5000 hospitals in the United States. This may engender a selection bias within the study population due to potential racial disparities in the utilization of high-volume CoC-accredited hospitals. Another caveat with the NCDB is its representation of mortality data, which pertains to ‘all-cause’ mortality rather than disease-specific mortality. Notably, these findings have not been externally validated, indicating the necessity for subsequent validation to strengthen the study’s conclusions.

This study demonstrates the utility of ML models in generating clinically useful individualized survival predictions for patients with WHO grade II and III gliomas. In contrast to traditional statistical approaches, these models can effectively capture intricate relationships and patterns in heterogeneous data that influence prognosis. The almost excellent discrimination exhibited by our models across multiple time points highlights their capability to provide precise, tailored forecasts that can inform clinical decision-making. The accessible web application represents a pioneering step in potentially integrating predictive analytics into routine neuro-oncology practice. By forecasting survival probabilities at specified intervals, this digital tool can inform patient-centered decision-making, risk stratification, and personalized management strategies. Clinicians can potentially utilize these prognostic estimates to have well-informed conversations with patients regarding likely outcomes, prioritize high-risk patients, and strategize care pathways accordingly. Overall, this work underscores the aptitude of machine learning for data-driven prognostication in neuro-oncology and demonstrates its immense potential to augment clinical care. The proposed models and web application address the challenges posed by the inherent heterogeneity of low-grade gliomas and pave the way for more precise, individualized prognostic estimates to optimize patient outcomes. Going forward, external validation of these models, along with impact analysis in clinical settings, will further elucidate the role of ML in advancing personalized medicine for neuro-oncology patients.

## Methods

**Fig. 5:**
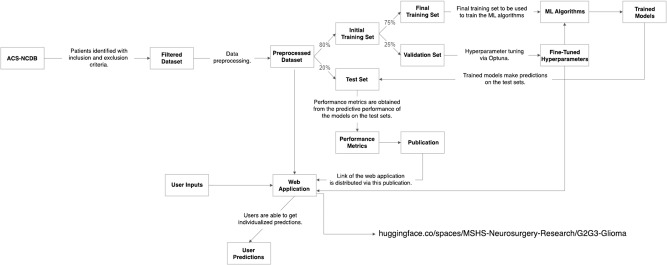
Flowchart of the study design (ACS-NCDB, American College of Surgeons – National Cancer Database; ML, machine learning).

The study design is summarized via a flowchart in Fig. 5.

### Ethical Approval

No institutional review board (IRB) approval or informed consent was required due to the use of de-identified patient data. The study was deemed exempt by the Icahn School of Medicine at Mount Sinai’s IRB.

### Data Source

The data for this study were sourced from the 2020 version of the NCDB. The NCDB is an expansive, prospectively maintained repository collaboratively developed by the CoC of the American College of Surgeons and the American Cancer Society. This sophisticated database encompasses information from over 1500 CoC-accredited institutions, accounting for approximately 70% of all cancer diagnoses within the United States, thereby providing an extensive breadth of data for analysis^35^.

### Guidelines

Transparent Reporting of Multivariable Prediction Models for Individual Prognosis or Diagnosis (TRIPOD)^36^ and Journal of Medical Internet Research (JMIR) Guidelines for Developing and Reporting Machine Learning Predictive Models in Biomedical Research^37^ were followed.

### Study Population

The NCDB-Brain Participant User File (PUF) was filtered for adults at least 18 years of age diagnosed with histologically confirmed cranial WHO grade II and III gliomas between 2010 and 2017. The initial temporal boundary of 2010 was chosen to reflect the advancements made in the treatment of gliomas over the preceding decade, whereas the terminal boundary of 2017 was selected to confine the study population. This latter limitation served to minimize the exclusion of patients due to the lack of extensive follow-up data. We used the International Classification of Disease for Oncology, third edition (ICD-O-3) histologic codes for diffuse astrocytoma [9400 (grade II)], anaplastic astrocytoma [9401 (grade III)], pleomorphic xanthoastrocytoma [9424 (grade II)], pilomyxoid astrocytoma [9425 (grade II)], oligodendroglioma [9450 (grade II)], anaplastic oligodendroglioma [9451 (grade III)], oligoastrocytoma [9382 (grade II)], and anaplastic oligoastrocytoma [9382 grade III)]; and ICD-O-3 topographical codes C71.0–C71.9 to define our patient population.

### Predictor Variables and Outcomes of Interest

Our study employed a range of predictor variables that span sociodemographic, clinicopathologic, and treatment-related attributes: 1) sociodemographics: age, sex, ethnicity, Spanish/Hispanic origin, primary payor, facility type, and facility location; 2) clinical presentation: Charlson-Deyo Score (as a measure of comorbidities), and Karnofsky Performance Scale; 3) diagnostic information: diagnostic biopsy (whether a diagnostic biopsy was taken before a possible resective surgery), tumor laterality, localization, focality (unifocal or multifocal), size (as ordinal), and histology; 4) molecular markers: 1p19q co-deletion, MGMT methylation, and Ki-67 labeling index; and 5) treatment modalities: resective surgery, extent of resection, radiation treatment, chemotherapy and immunotherapy. Ethnicity and Spanish/Hispanic origin variables were collected and reported by the NCDB, along with other variables. For detailed information regarding the data items a data dictionary can be found at https://www.facs.org/media/brilfbgu/puf-2020-data-dictionary.pdf. For categorical variables, the missing values were filled with ‘Unknown’ or ‘Unknown/Other’. The only continuous variable in the data, age, had no missing values.

We built separate prediction models to predict patient survival outcomes for WHO grade II and grade III glioma patients at four distinct time points post-diagnosis: 12, 24, 36, and 60 months. The mortality outcomes were extrapolated by combining ‘Vital Status’ and ‘Last Contact or Death (Months from Diagnosis)’ variables. Patients with missing data in the ‘Vital Status’ and ‘Last Contact or Death (Months from Diagnosis)’ data items were excluded. To elaborate, when identifying patients who died within 12 months following their diagnosis, we filtered our data for cases where the ‘Vital Status’ was recorded as ‘Dead’, with the ‘Last Contact or Death (Months from Diagnosis)’ being less than twelve months. These individuals have then ascribed a 12-month mortality status of ‘Yes’. Conversely, patients with a ‘Vital Status’ listed as ‘Alive’ and a ‘Last Contact or Death (Months from Diagnosis)’ beyond 12 months or ‘Vital Status’ listed as ‘Dead’ but ‘Last Contact or Death (Months from Diagnosis)’ was more than 12 months were given a 12-month mortality status of ‘No’. In cases where a patient was logged as alive, but their latest follow-up data [‘Last Contact or Death (Months from Diagnosis)’] was recorded prior to the specific survival time point in question, they were omitted from the pertinent survival analyses. We followed the same approach for survival outcomes at 24, 36, and 60 months for both grade II and grade III patients.

### Model Development and Evaluation

In this study, five distinct supervised ML algorithms were employed: TabPFN, TabNet, XGBoost, LightGBM, and Random Forest. Predictive models built with supervised ML algorithms are trained to utilize data sets for which the actual outcomes are known. These models can then assimilate this data, allowing for the provision of accurate predictions when encountering new, unexplored data. Each of these algorithms was selected due to its unique abilities and has demonstrated a high-level performance in differentiating or categorizing data, the capability to handle a multitude of variables simultaneously, and versatility in adjustments.

TabPFN represents a type of transformer-based algorithm that demonstrates the ability to decode complex patterns within point cloud data, which are essentially data points organized in a spatial configuration^38^. TabNet is an exemplification of a deep learning model that offers a self-explanatory and interpretation-oriented structure, competent in managing various forms of structured data^39^. Both XGBoost and LightGBM are gradient-boosting frameworks that exhibit high efficiency in addressing classification problems^20,40^. The Random Forest algorithm operates by constructing a multitude of decision trees, with the final decision being derived from the collective decision of these trees^21^.

For each of these algorithms (except TabPFN), hyperparameter optimization was carried out employing the Optuna library^41^. Optuna, an adaptable framework for hyperparameter optimization, enables the automatic adaptation of parameters. The purpose of this optimization process was to identify the parameters that produce the model with the highest performance, as indicated by the AUROC. The hyperparameter spaces for each are provided in Supplementary Table 3.

To guarantee ample data for the stages of model development, validation, and evaluation, the data set for each of the outcomes of interest were divided into three subsets using a 60:20:20 distribution for training, validation, and test sets, respectively. The training sets were used to train the ML models, the validations set were utilized for hyperparameter tuning and calibration, and the test sets were used to evaluate the models’ performance.

Before the initiation of model training, the Synthetic Minority Over-sampling Technique (SMOTE) was applied to the training sets^42^. SMOTE addresses any imbalanced class distributions within a dataset by generating artificial samples from the lesser-represented class, thus increasing the instances of samples in the under-represented class rather than replicating existing samples. This method effectively enhances the volume of samples in the under-represented class, hence increasing the performance of the ML models.

The performances of the models were evaluated both visually and numerically. The visual assessment was completed using the ROC and PRC. The ROCs, graphically, are designed to represent the diagnostic proficiency of binary classifiers throughout a range of discrimination thresholds^43^. They achieve this by plotting the ‘true positive rate’ [*true positives (TP)/(TP+false negatives [FN])*] against the ‘false positive rate’ [*FP/(TP* + *FP)*] at a spectrum of threshold settings, thereby providing a consolidated indicator of performance irrespective of the classification thresholds. PRCs are graphical demonstrations of ‘precision’ [also known as positive predictive value, *TP/(TP* + *FP)*] juxtaposed against ‘recall’ [which is the same as true positive rate or sensitivity, *TP/(TP* + *FN)*]. These curves are deemed especially valuable when working with datasets that exhibit a class imbalance due to their ability to elucidate the trade-off existing between precision and recall at various thresholds.

The numerical evaluation involved metrics such as sensitivity, specificity, accuracy, area under the PRC (AUPRC), and AUROC. Furthermore, we assessed the calibration of our models utilizing the Brier score, which is the average squared difference between predicted and actual probabilities^44,45^. A well-calibrated model will have a Brier score close to zero, indicating no difference between the predicted and actual probabilities. Confusion matrices were also generated for interpreting the performance of our models by presenting a clear depiction of correct and incorrect classifications made.

Top-performing models were selected for deployment in our web-based application according to their AUROC scores. AUROC is a widely accepted performance indicator for ML models, particularly pertinent in binary classification tasks^46^. It quantifies a model’s ability to discern between positive and negative instances across various classification thresholds. The utility of AUROC as our primary metric is threefold. First, it is unaffected by class imbalance, making it suitable for datasets with uneven class distributions. Second, it takes into consideration all classification thresholds, offering a comprehensive evaluation of model performance at different points. Third, by encapsulating the model’s performance into a singular score, AUROC simplifies the comparison of different models or algorithms. Therefore, it provides a reliable representation of the model’s ability to differentiate, thus rendering it a suitable metric for model evaluation and selection across various applications.

To enhance the interpretability of our models, we employed SHAP to ascertain the relative significance of predictor variables^47^. Additionally, PDPs were utilized to illustrate the impact of individual variables on the predictions made by the top-performing models^48^.

### Web Application

We developed a web application that empowers healthcare professionals to generate predictions for individual patients. The top-performing models, according to their AUROC scores as explained above, for each outcome have been incorporated into this application. The hyperparameters used in the web application are provided in Supplementary Table 4. Hugging Face serves as a platform that promotes the sharing of ML models among individuals and is where our implementation code for these models is accessible. The functionality of the web application is demonstrated in a video (Supplementary Video 1). It can be reached at this URL: https://huggingface.co/spaces/MSHS-Neurosurgery-Research/G2G3-Glioma.

### Descriptive Statistics

Descriptive statistics were reported as means (with ± standard deviations) for continuous variables with normal distributions, medians (with interquartile ranges) for non-normally distributed continuous variables, and numbers (with % percentages) for categorical variables. Differences between grade II and grade III patient cohorts were tested using independent *t*-tests for normally distributed continuous variables with equal variances, Welch’s *t*-tests for normally distributed continuous variables with unequal variances, Mann-Whitney U tests for non-normally distributed continuous variables, and Pearson’s chi-squared tests for categorical variables. Normality was assessed using the Shapiro-Wilk test. Levene’s test evaluated the equality of variances. Differences were considered statistically significant at *p* < 0.001.

### Reporting summary

Further information on research design is available in the Nature Research Reporting Summary linked to this article.

### Supplementary information

Supplementary Information

Supplementary Video 1

Reporting Summary

## Data Availability

Restrictions apply to the availability of the data. Data were obtained from the NCDB, a prospectively maintained repository collaboratively developed by the CoC of the American College of Surgeons and the American Cancer Society. None of these institutions have verified and are not responsible for the statistical validity of the data analysis or the conclusions derived by the authors.

## Code availability

The source code for preprocessing and analyzing the data is available on GitHub (https://github.com/mertkarabacak/NCDB-G2G3_Glioma).
