# Supplementary material for: Prognosis Individualized: Survival predictions for WHO grade II and III gliomas with a machine learning-based web application
Source: NPJ Digit Med. 2023 Oct 26;6:200. doi: 10.1038/s41746-023-00948-y (PMC10603035; doi:10.1038/s41746-023-00948-y)
Supplement: Supplementary file 1 — Supplementary Information [file 41746_2023_948_MOESM1_ESM.pdf]

Supplementary Figure 1. Confusion matrices for a) the Random Forest model predicting 12-month mortality, b) the LightGBM model predicting 24-month mortality, c) the LightGBM model predicting 36-month mortality, d) the Random Forest model predicting 60-month mortality for WHO grade II gliomas; and e) the LightGBM model predicting 12-month mortality, f) the Random Forest model predicting 24-month mortality, g) the Random Forest model predicting 36-month mortality, h) the LightGBM model predicting 60-month mortality for WHO grade III gliomas.

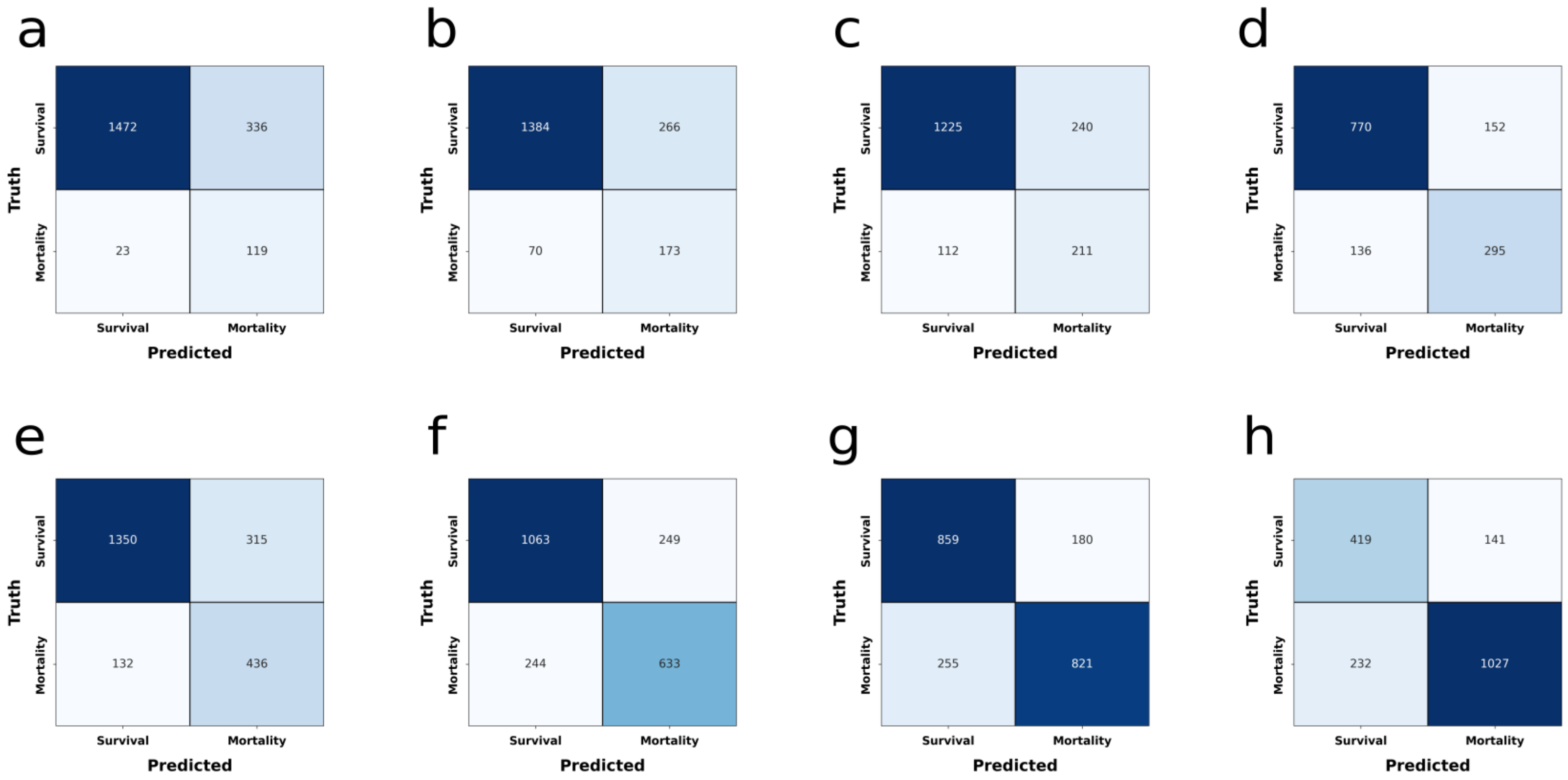

Supplementary Figure 2. Confusion matrices for the models predicting the outcome at 12-month mortality for WHO grade II gliomas with the a) TabPFN, b) TabNet, c) XGBoost, and d) LightGBM algorithms.

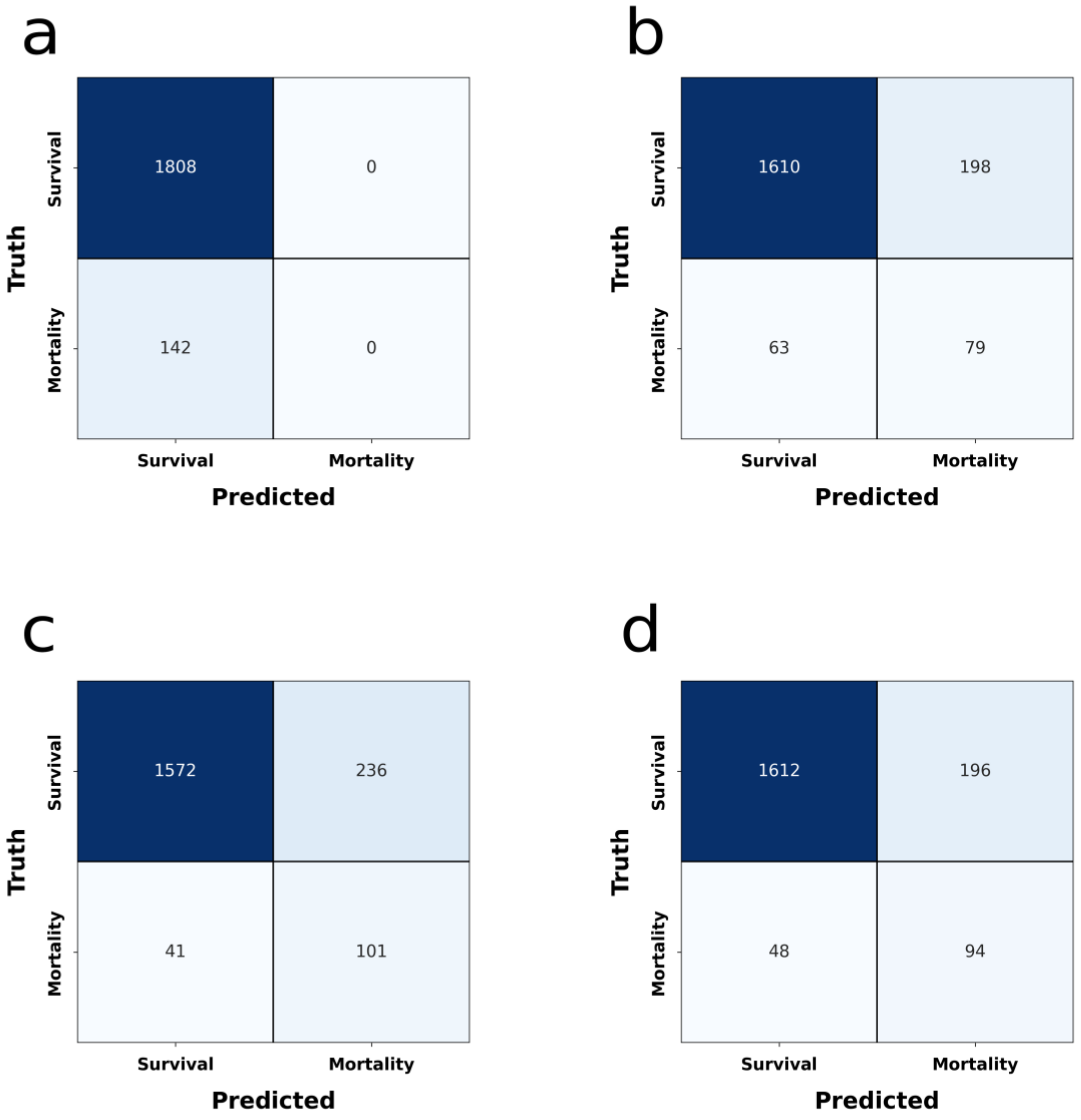

Supplementary Figure 3. Confusion matrices for the models predicting the outcome at 24-month mortality for WHO grade II gliomas with the a) TabPFN, b) TabNet, c) XGBoost, and d) Random Forest algorithms.

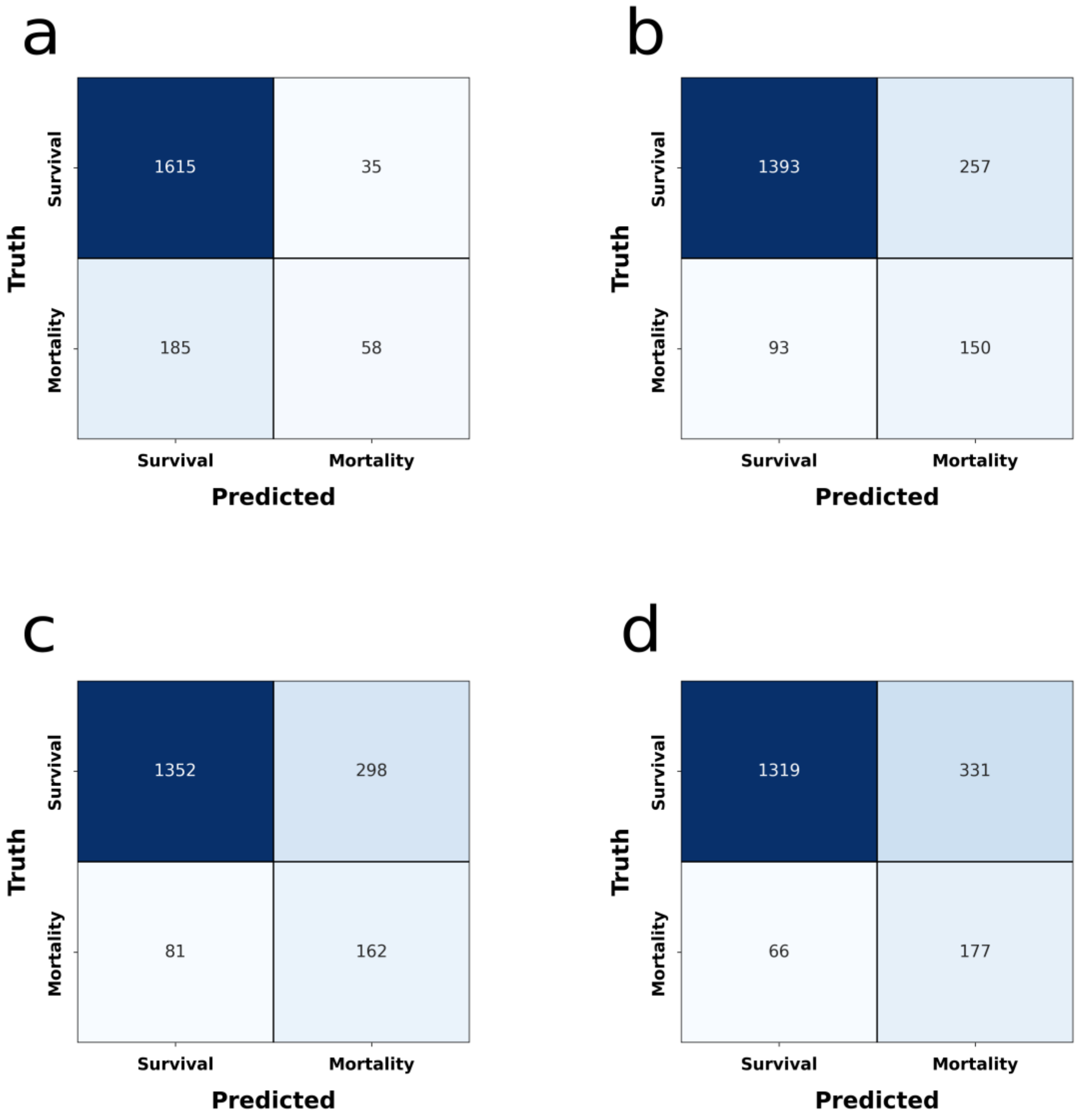

Supplementary Figure 4. Confusion matrices for the models predicting the outcome at 36-month mortality for WHO grade II gliomas with the a) TabPFN, b) TabNet, c) XGBoost, and d) Random Forest algorithms.

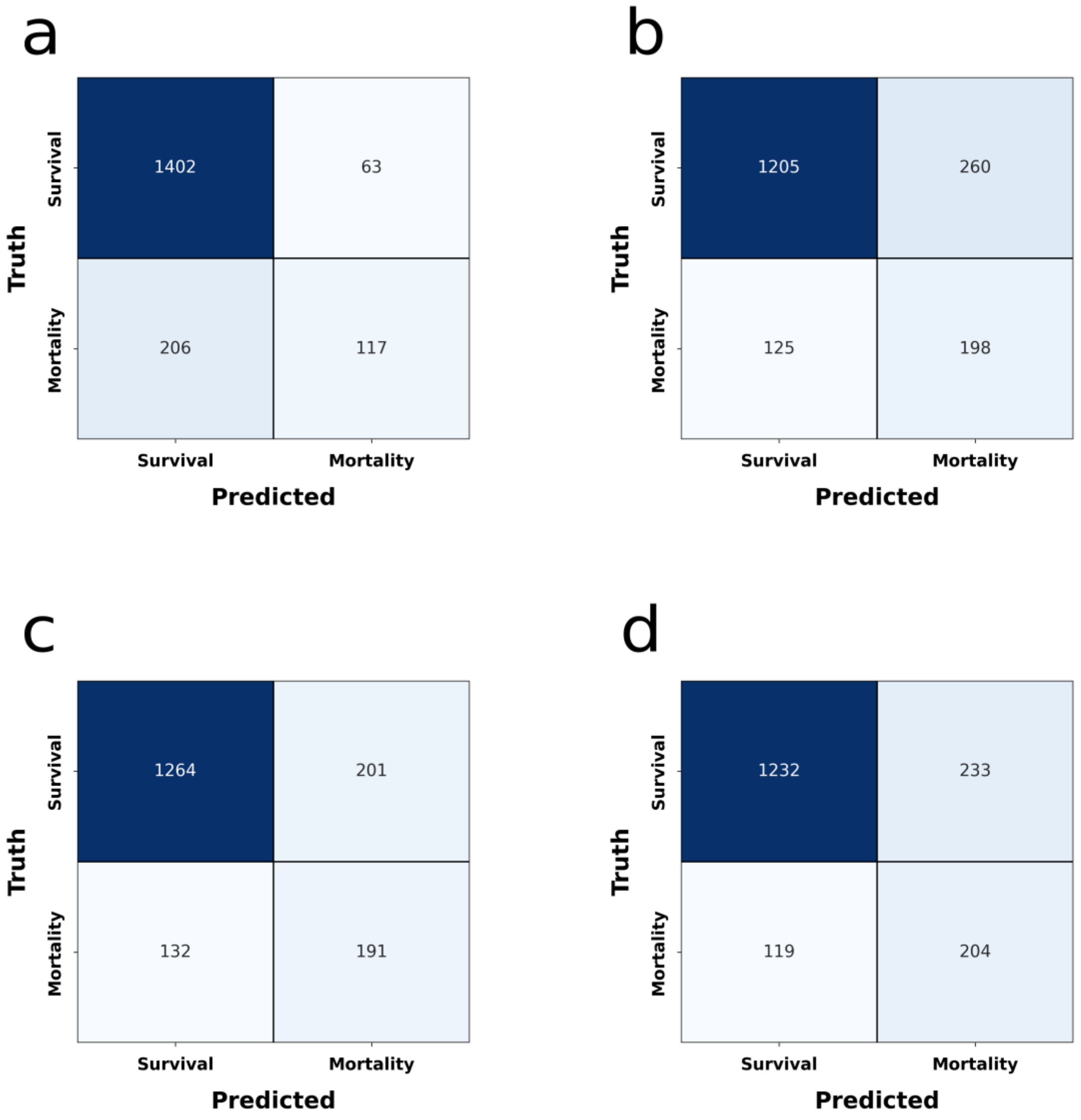

Supplementary Figure 5. Confusion matrices for the models predicting the outcome at 60-month mortality for WHO grade II gliomas with the a) TabPFN, b) TabNet, c) XGBoost, and d) LightGBM algorithms.

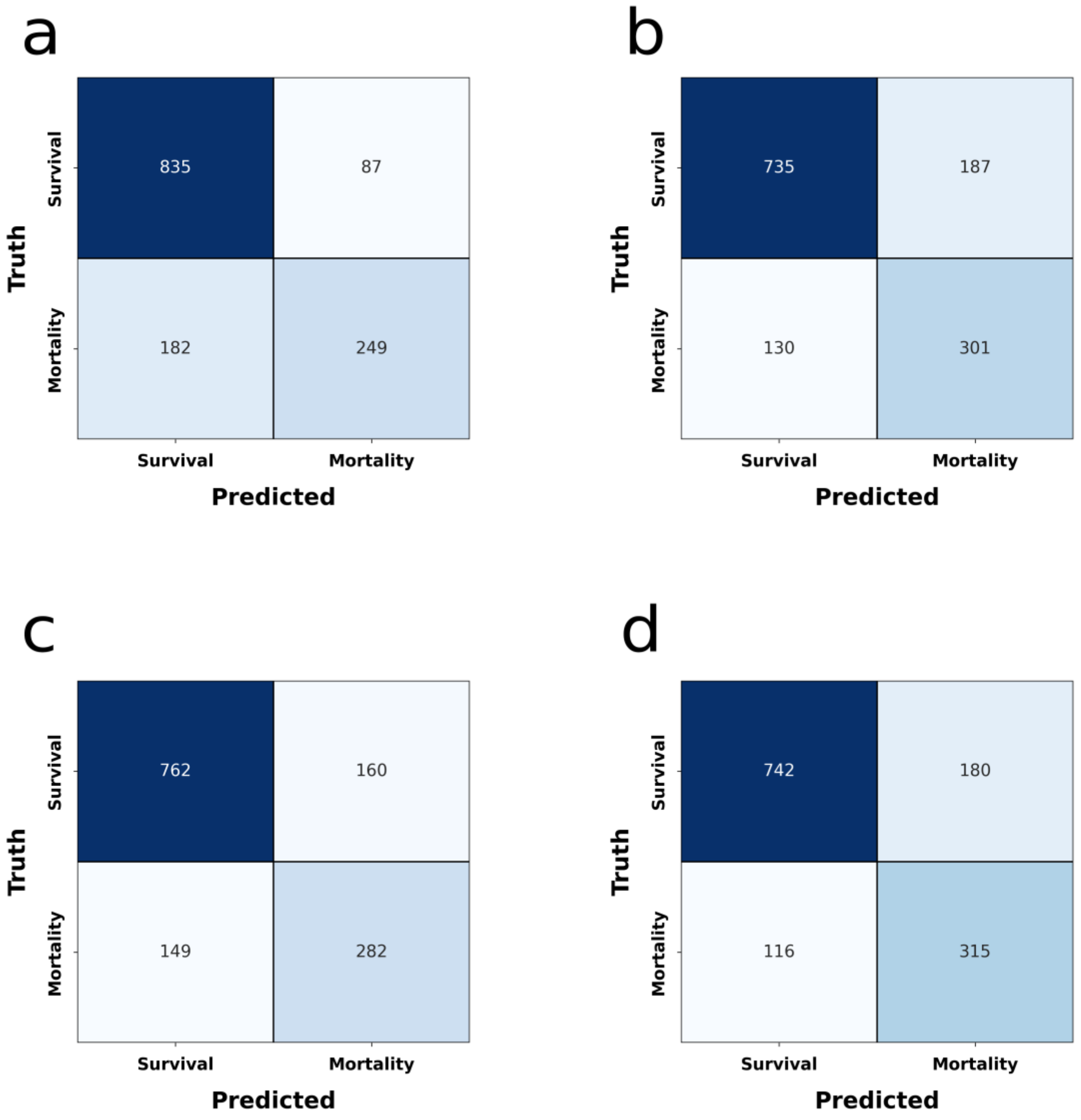

Supplementary Figure 6. Confusion matrices for the models predicting the outcome at 12-month mortality for WHO grade III gliomas with the a) TabPFN, b) TabNet, c) XGBoost, and d) Random Forest algorithms.

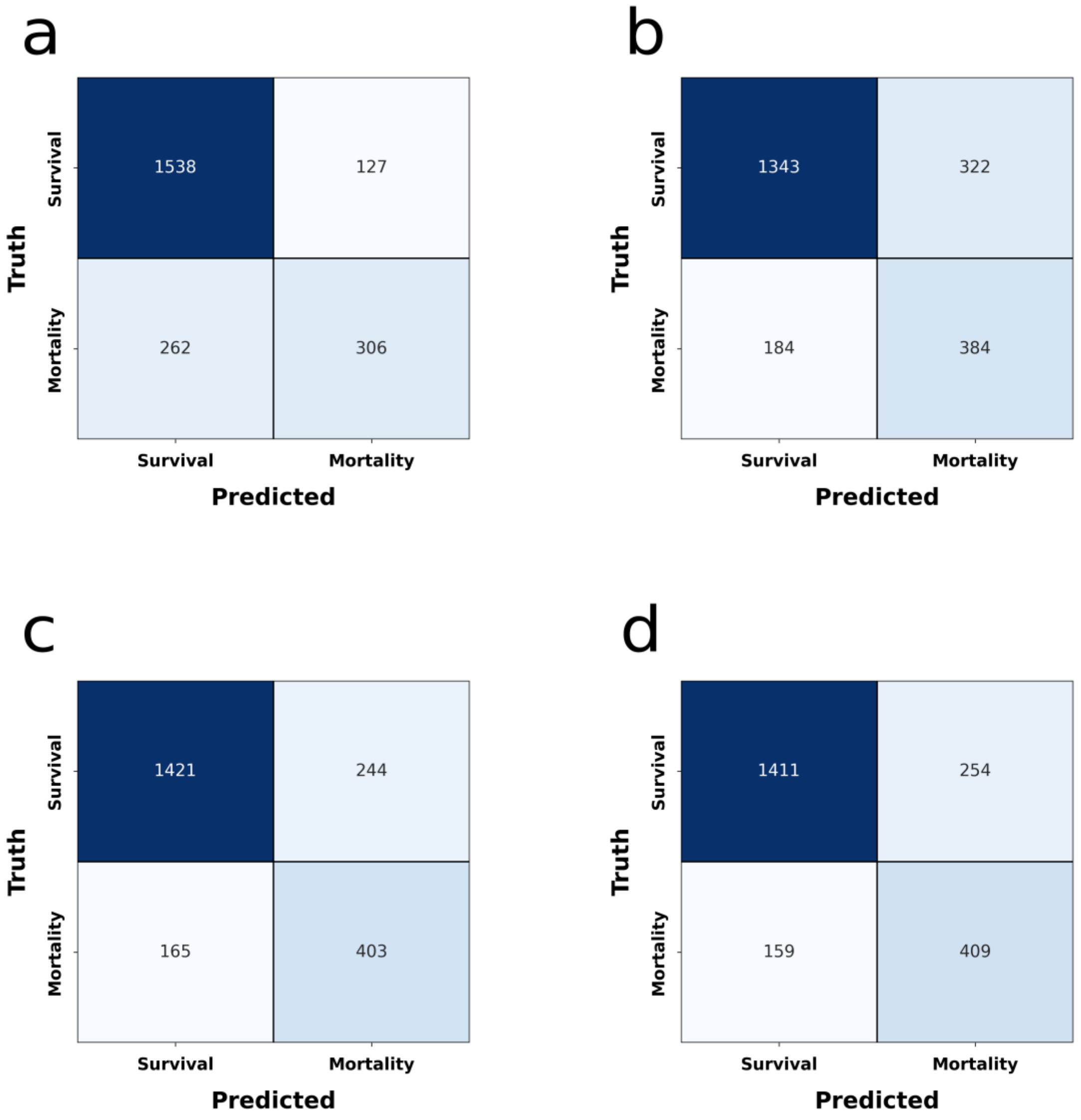

Supplementary Figure 7. Confusion matrices for the models predicting the outcome at 24-month mortality for WHO grade III gliomas with the a) TabPFN, b) TabNet, c) XGBoost, and d) LightGBM algorithms.

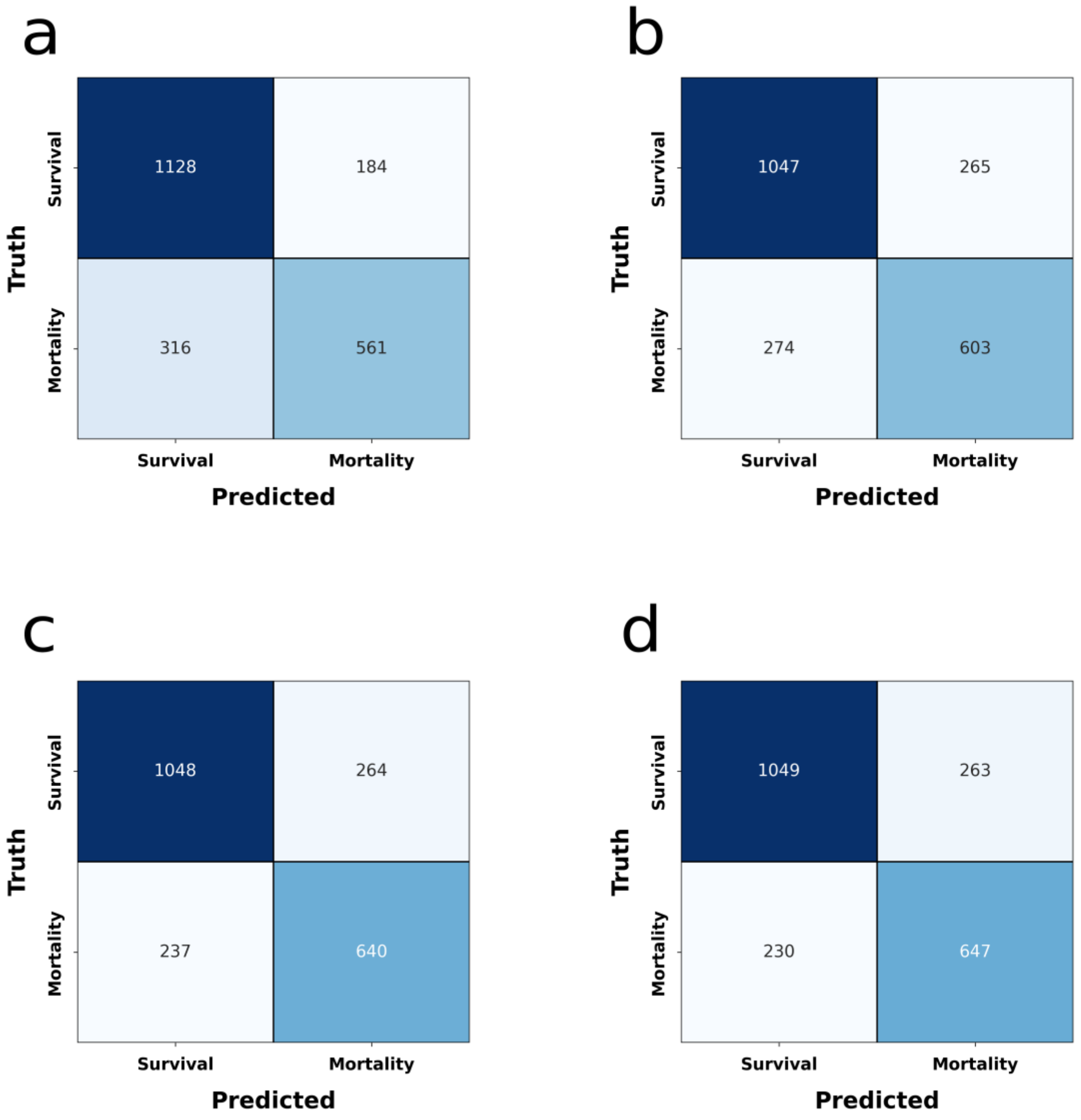

Supplementary Figure 8. Confusion matrices for the models predicting the outcome at 36-month mortality for WHO grade III gliomas with the a) TabPFN, b) TabNet, c) XGBoost, and d) LightGBM algorithms.

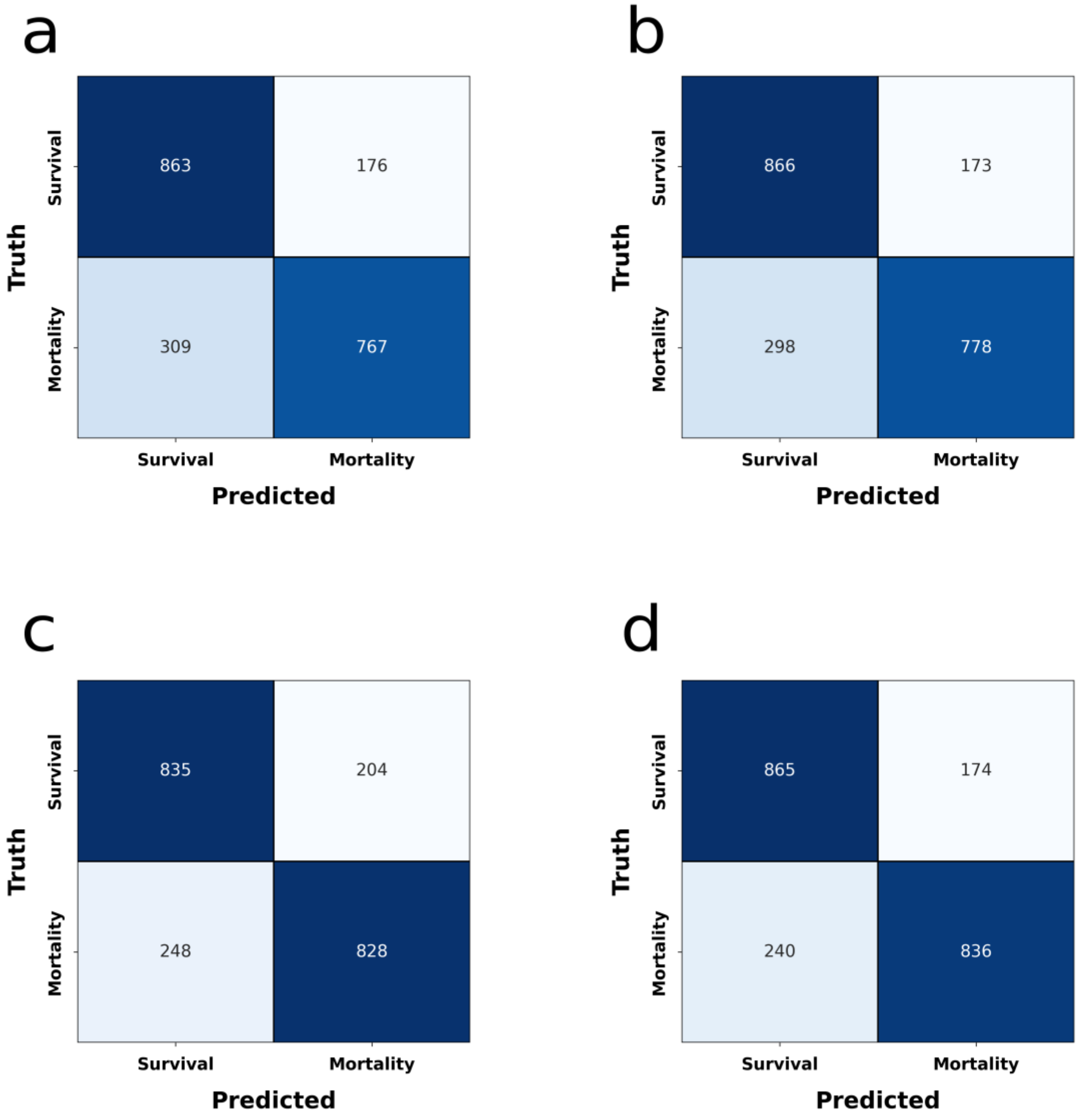

Supplementary Figure 9. Confusion matrices for the models predicting the outcome at 60-month mortality for WHO grade III gliomas with the a) TabPFN, b) TabNet, c) XGBoost, and d) Random Forest algorithms.

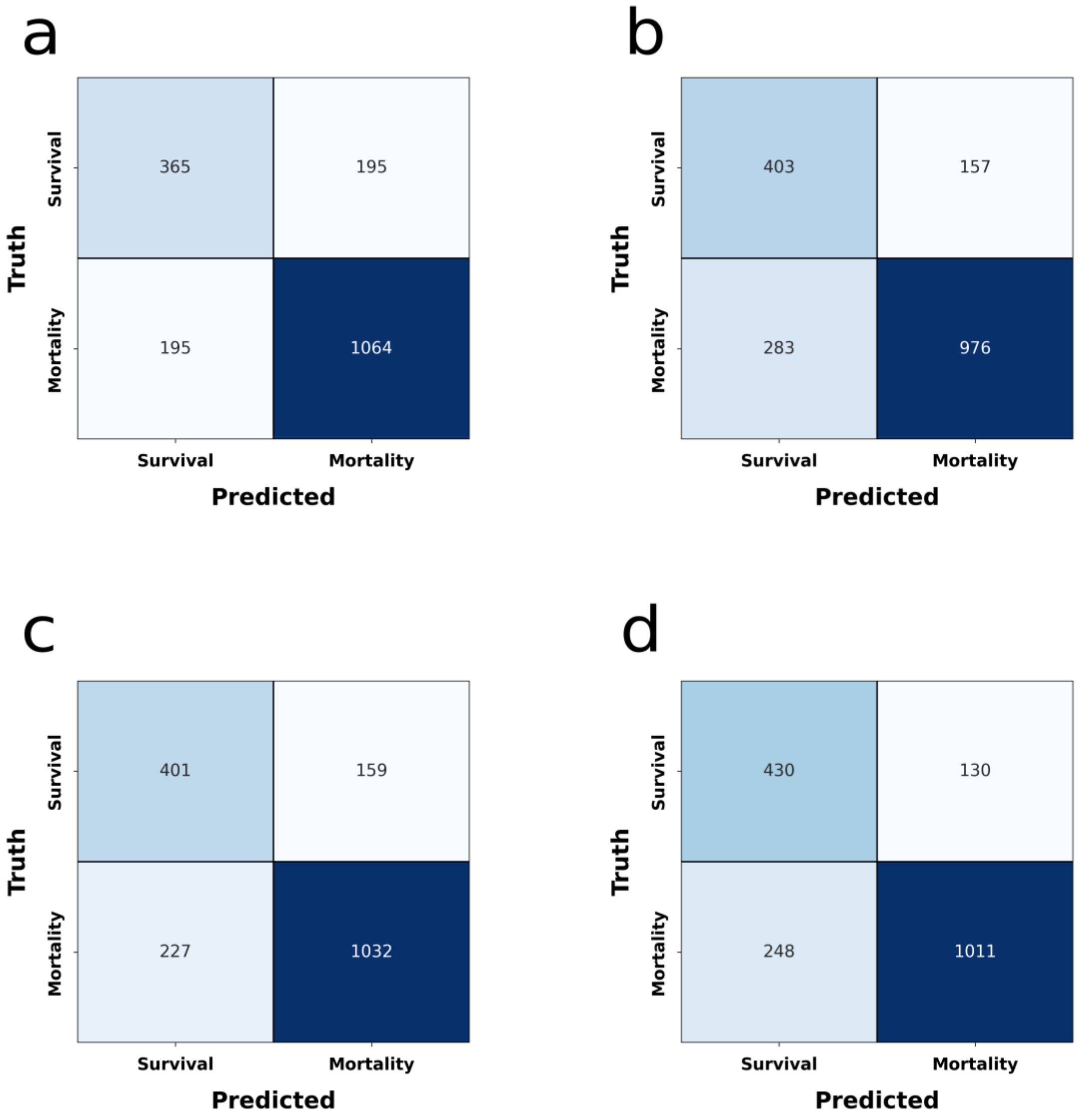

Supplementary Figure 10. The 15 most important features and their mean SHapley Additive exPlanations (SHAP) values for the models predicting the outcome at 12-month mortality for WHO grade II gliomas with the a) TabPFN, b) TabNet, c) XGBoost, and d) LightGBM algorithms (SHAP, SHapley Additive exPlanations).

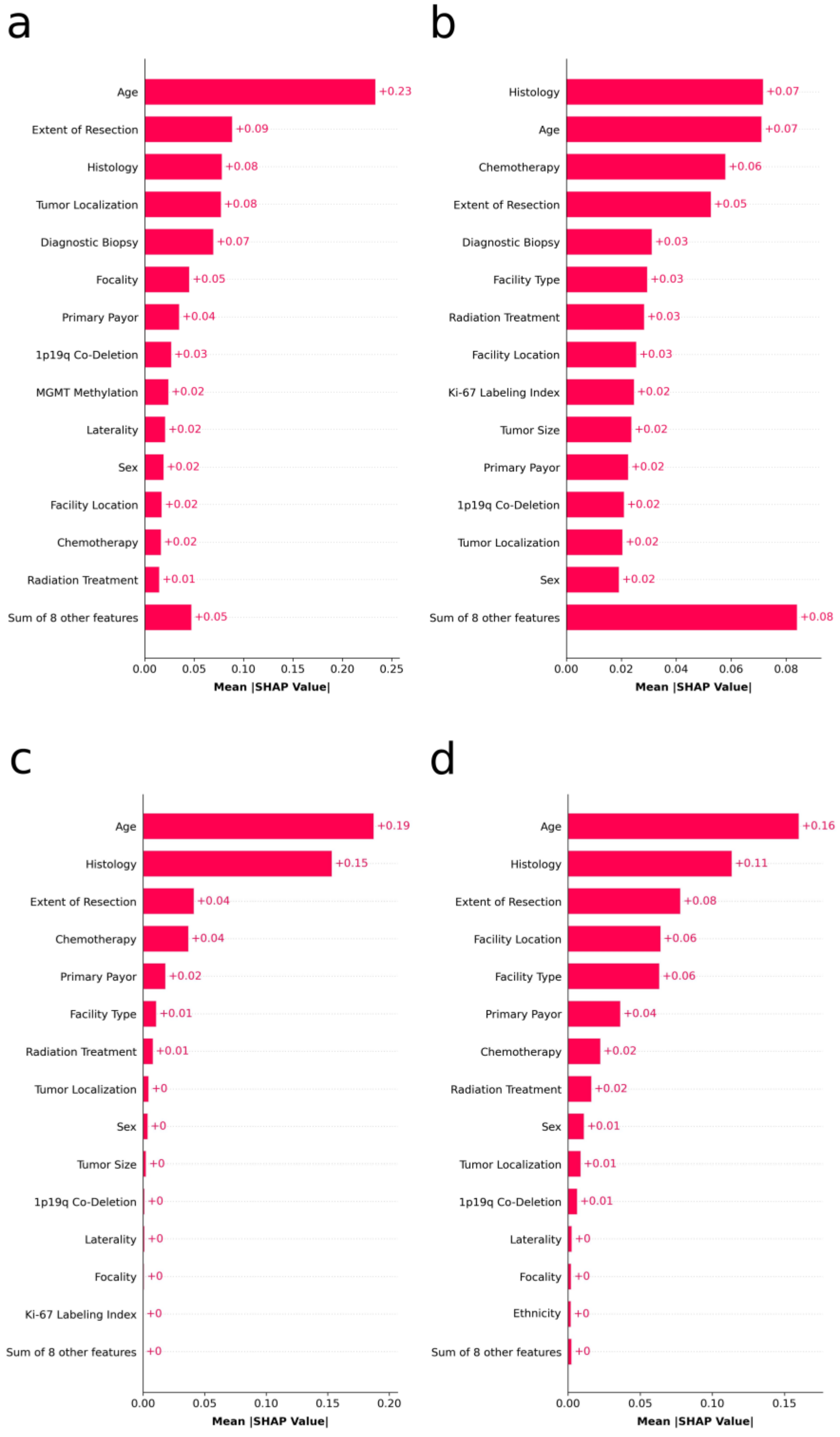

Supplementary Figure 11. The 15 most important features and their mean SHapley Additive exPlanations (SHAP) values for the models predicting the outcome at 24-month mortality for WHO grade II gliomas with the a) TabPFN, b) TabNet, c) XGBoost, and d) Random Forest algorithms (SHAP, SHapley Additive exPlanations).

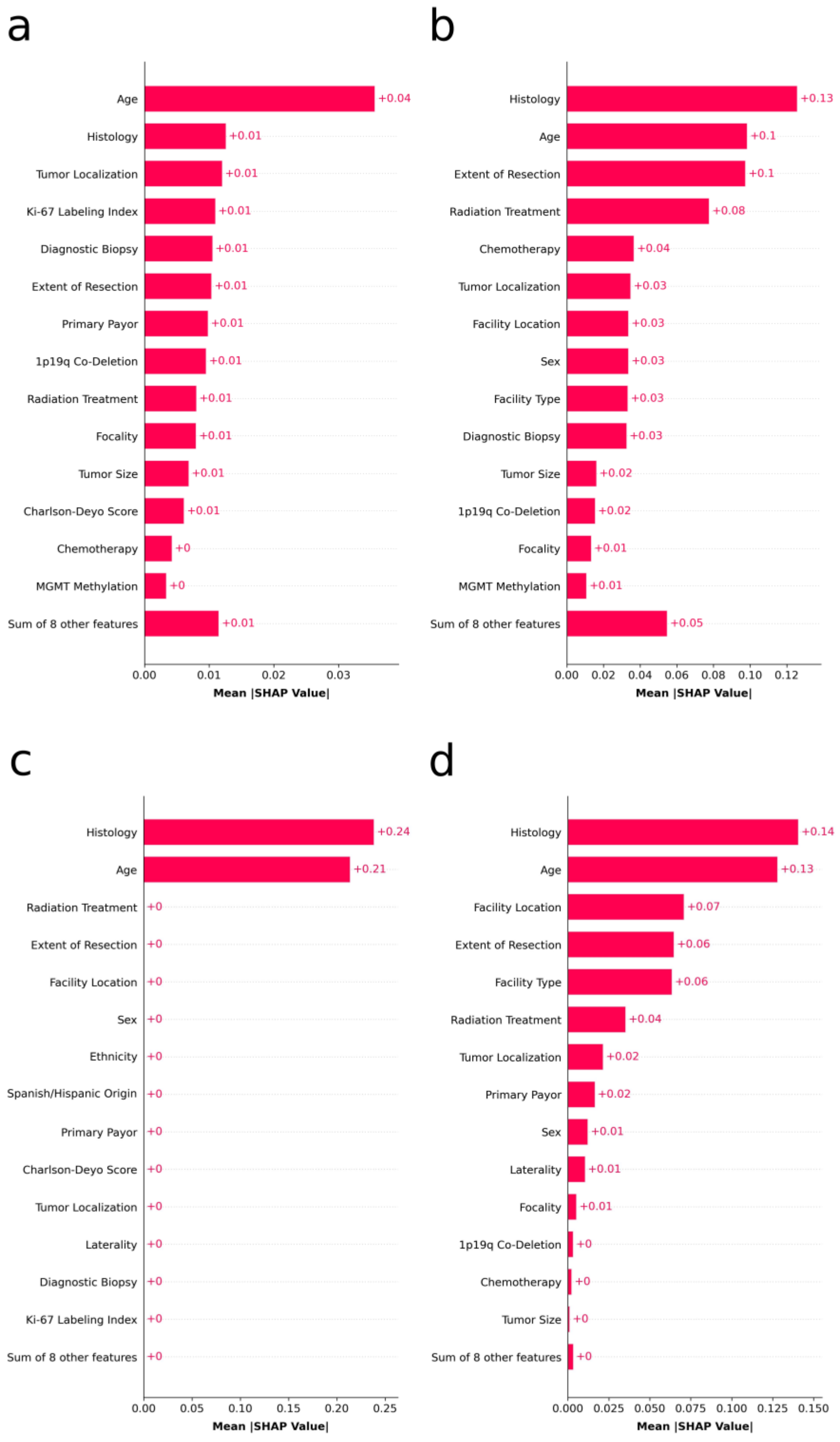

Supplementary Figure 12. The 15 most important features and their mean SHapley Additive exPlanations (SHAP) values for the models predicting the outcome at 36-month mortality for WHO grade II gliomas with the a) TabPFN, b) TabNet, c) XGBoost, and d) Random Forest algorithms (SHAP, SHapley Additive exPlanations).

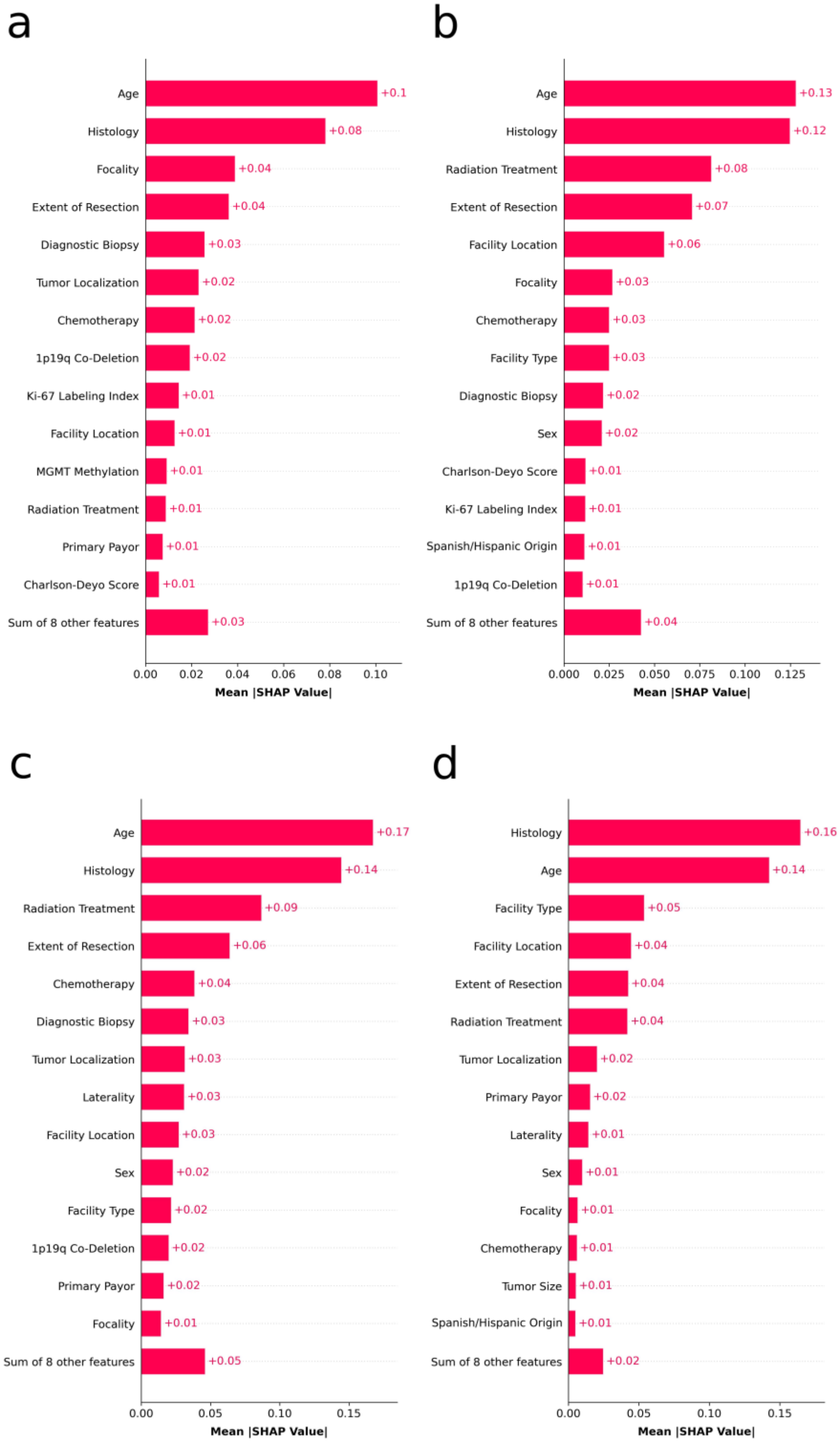

Supplementary Figure 13. The 15 most important features and their mean SHapley Additive exPlanations (SHAP) values for the models predicting the outcome at 60-month mortality for WHO grade II gliomas with the a) TabPFN, b) TabNet, c) XGBoost, and d) LightGBM algorithms (SHapley Additive exPlanations).

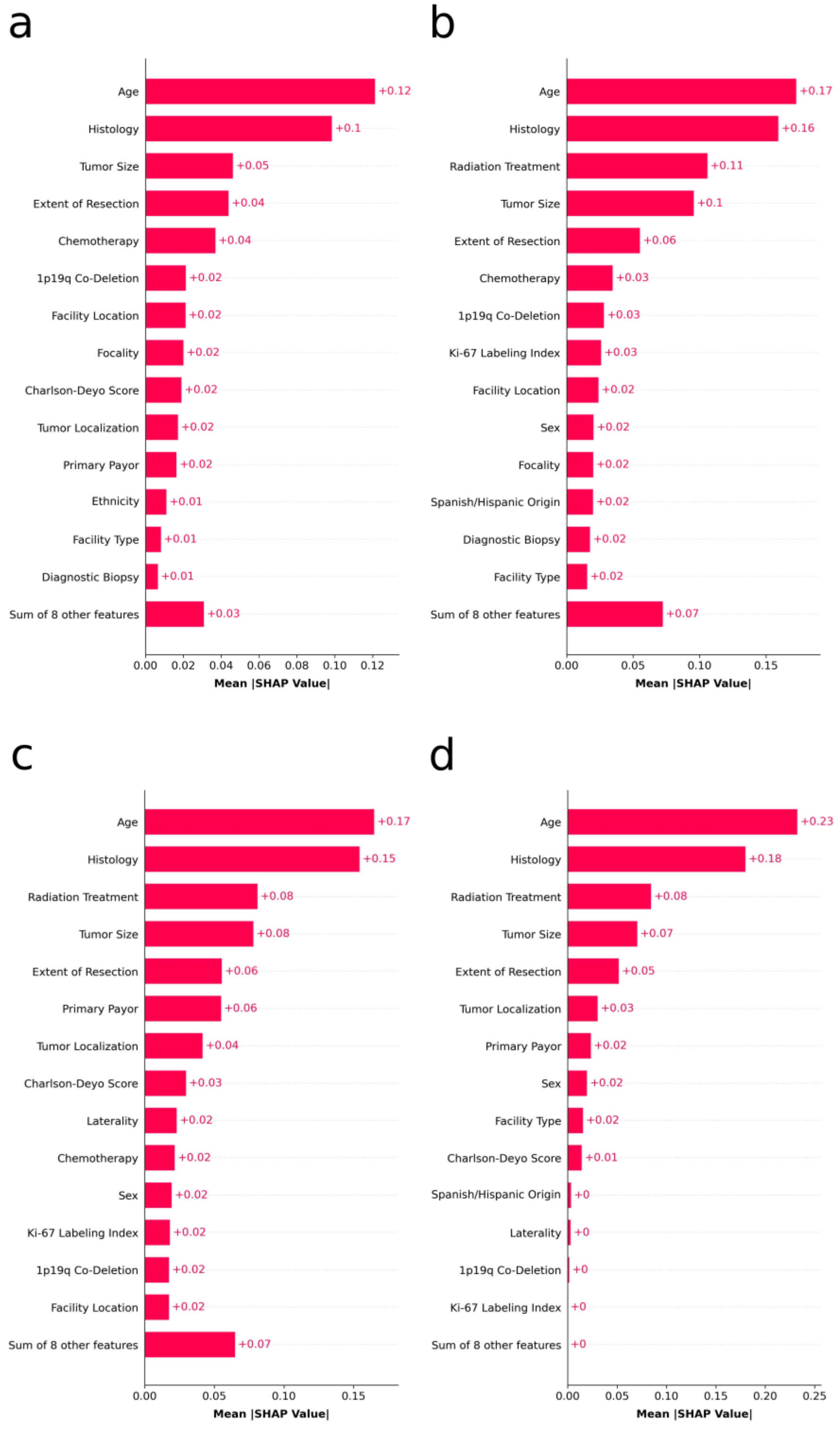

Supplementary Figure 14. The 15 most important features and their mean SHapley Additive exPlanations (SHAP) values for the models predicting the outcome at 12-month mortality for WHO grade III gliomas with the a) TabPFN, b) TabNet, c) XGBoost, and d) Random Forest algorithms (SHAP, SHapley Additive exPlanations).

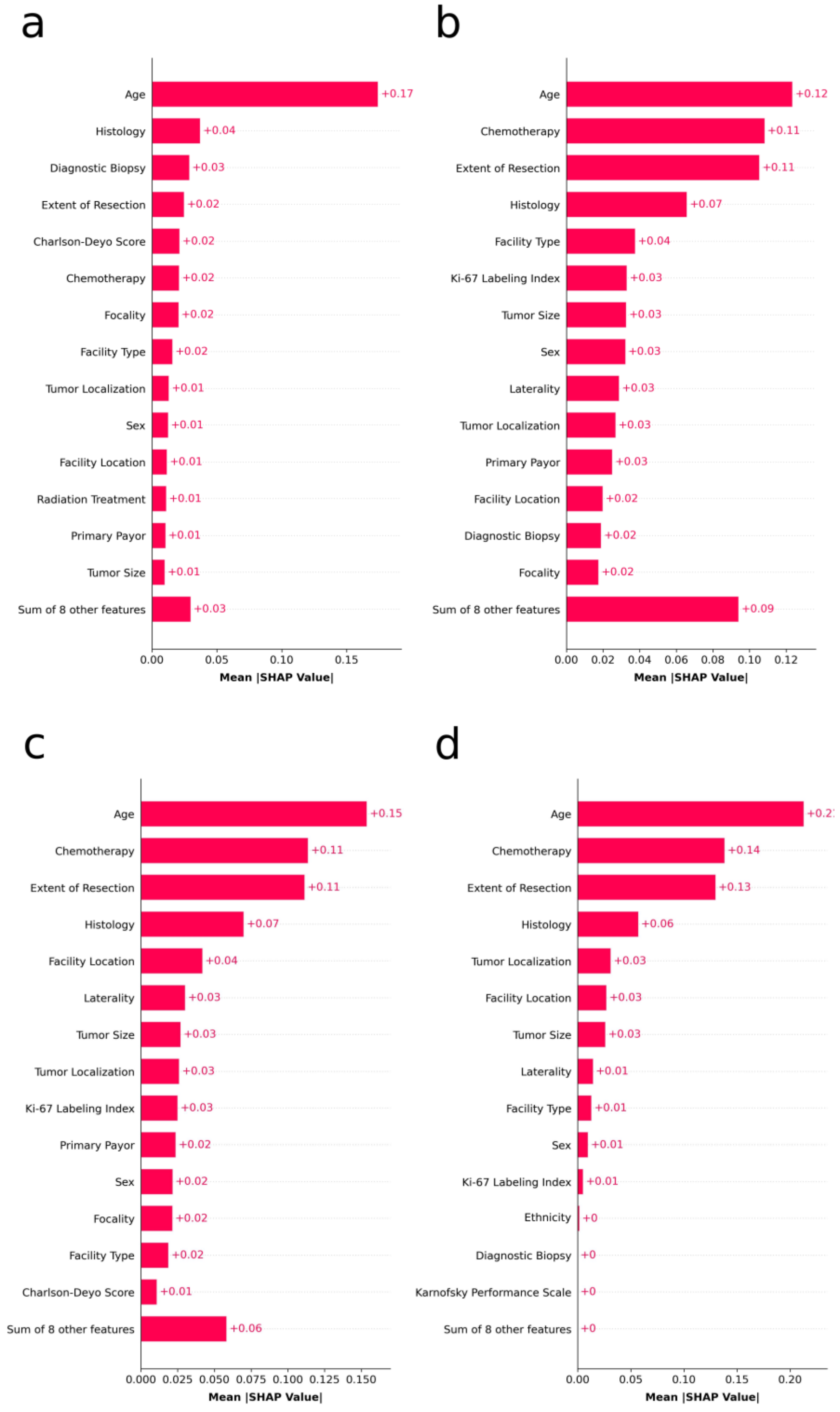

Supplementary Figure 15. The 15 most important features and their mean SHapley Additive exPlanations (SHAP) values for the models predicting the outcome at 24-month mortality for WHO grade III gliomas with the a) TabPFN, b) TabNet, c) XGBoost, and d) LightGBM algorithms (SHAP, SHapley Additive exPlanations).

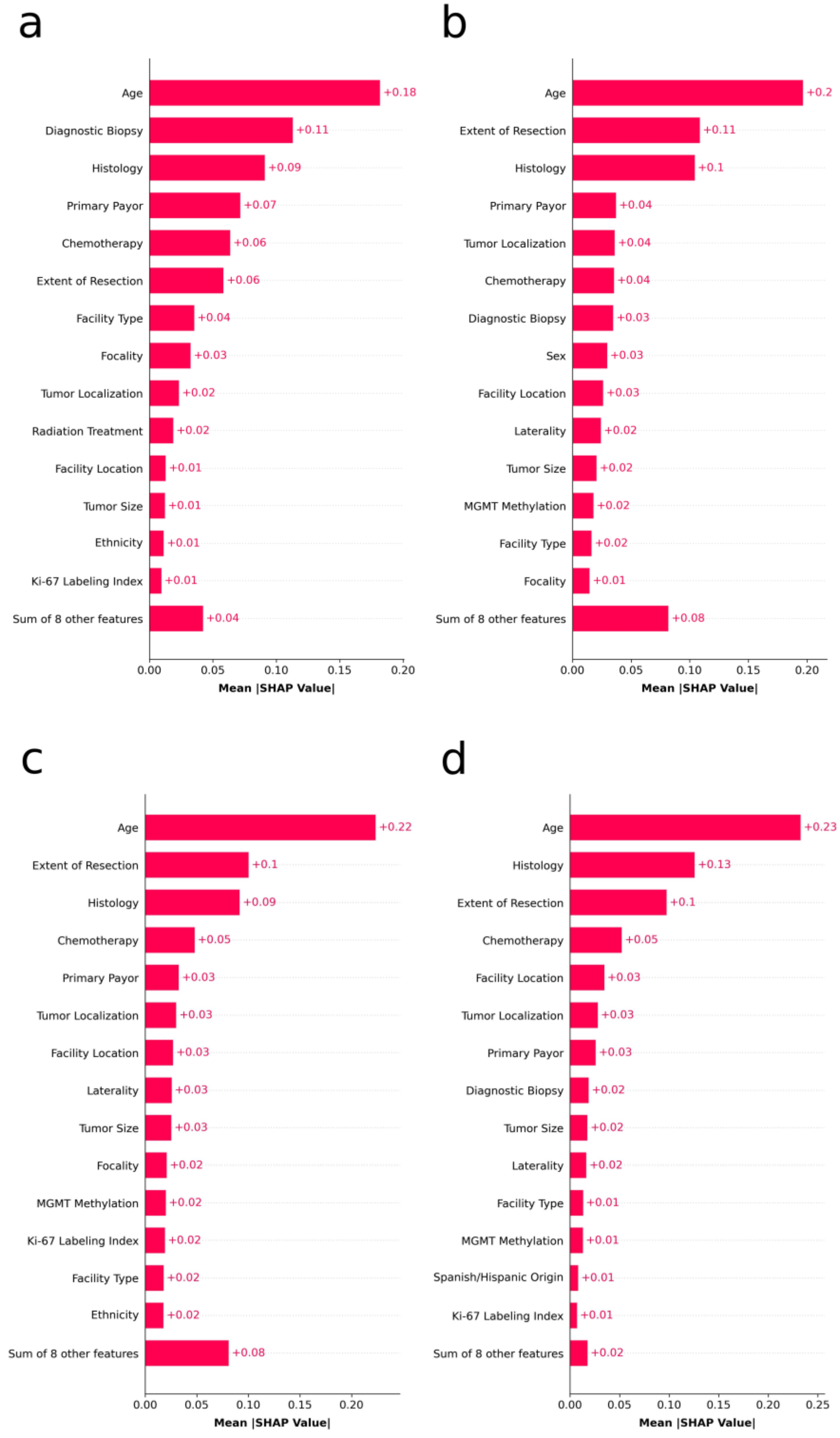

Supplementary Figure 16. The 15 most important features and their mean SHapley Additive exPlanations (SHAP) values for the models predicting the outcome at 36-month mortality for WHO grade III gliomas with the a) TabPFN, b) TabNet, c) XGBoost, and d) LightGBM algorithms (SHAP, SHapley Additive exPlanations).

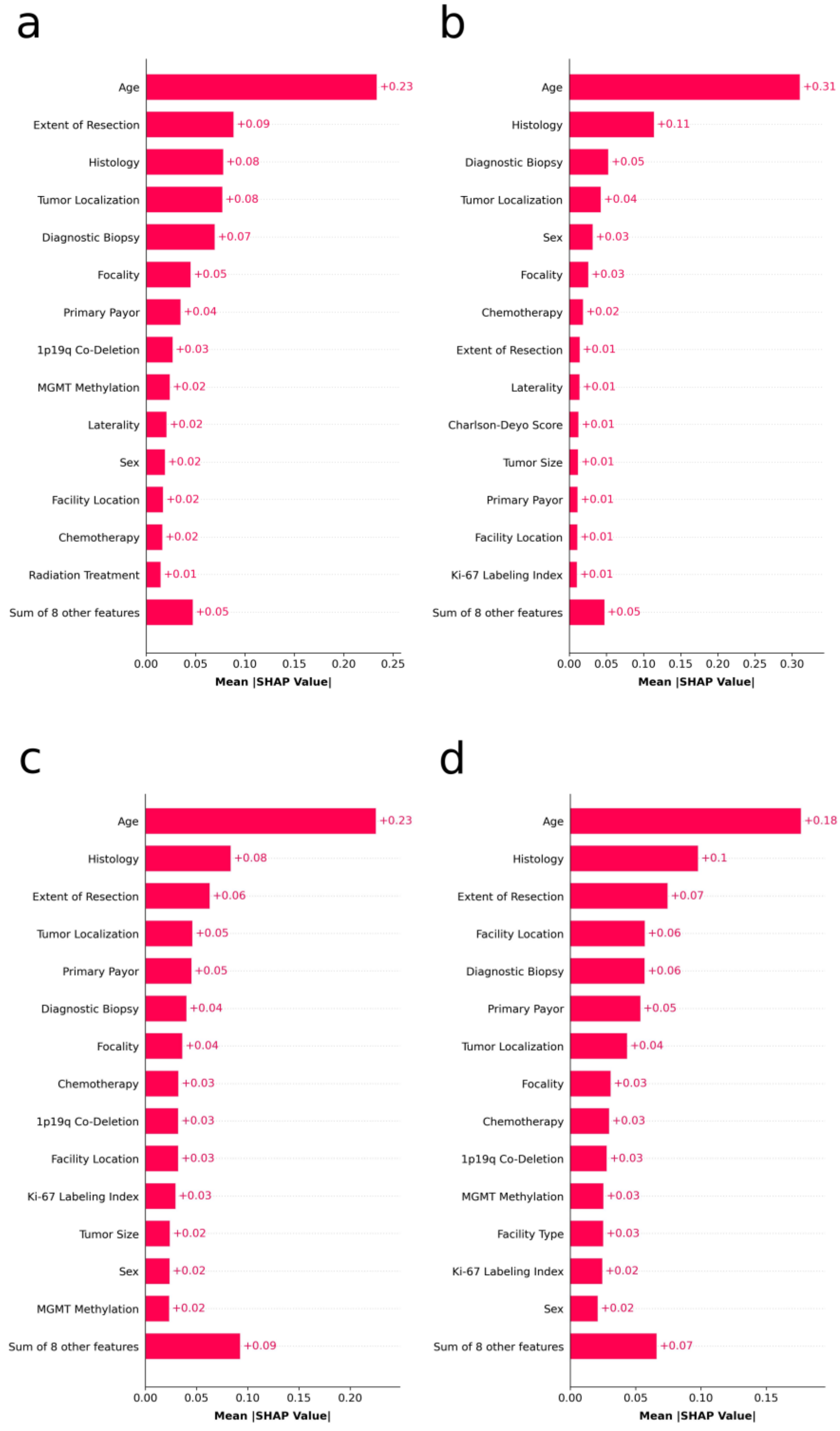

Supplementary Figure 17. The 15 most important features and their mean SHapley Additive exPlanations SHAP values for the models predicting the outcome at 60-month mortality for WHO grade III gliomas with the a) TabPFN, b) TabNet, c) XGBoost, and d) Random Forest algorithms (SHAP, SHapley Additive exPlanations).

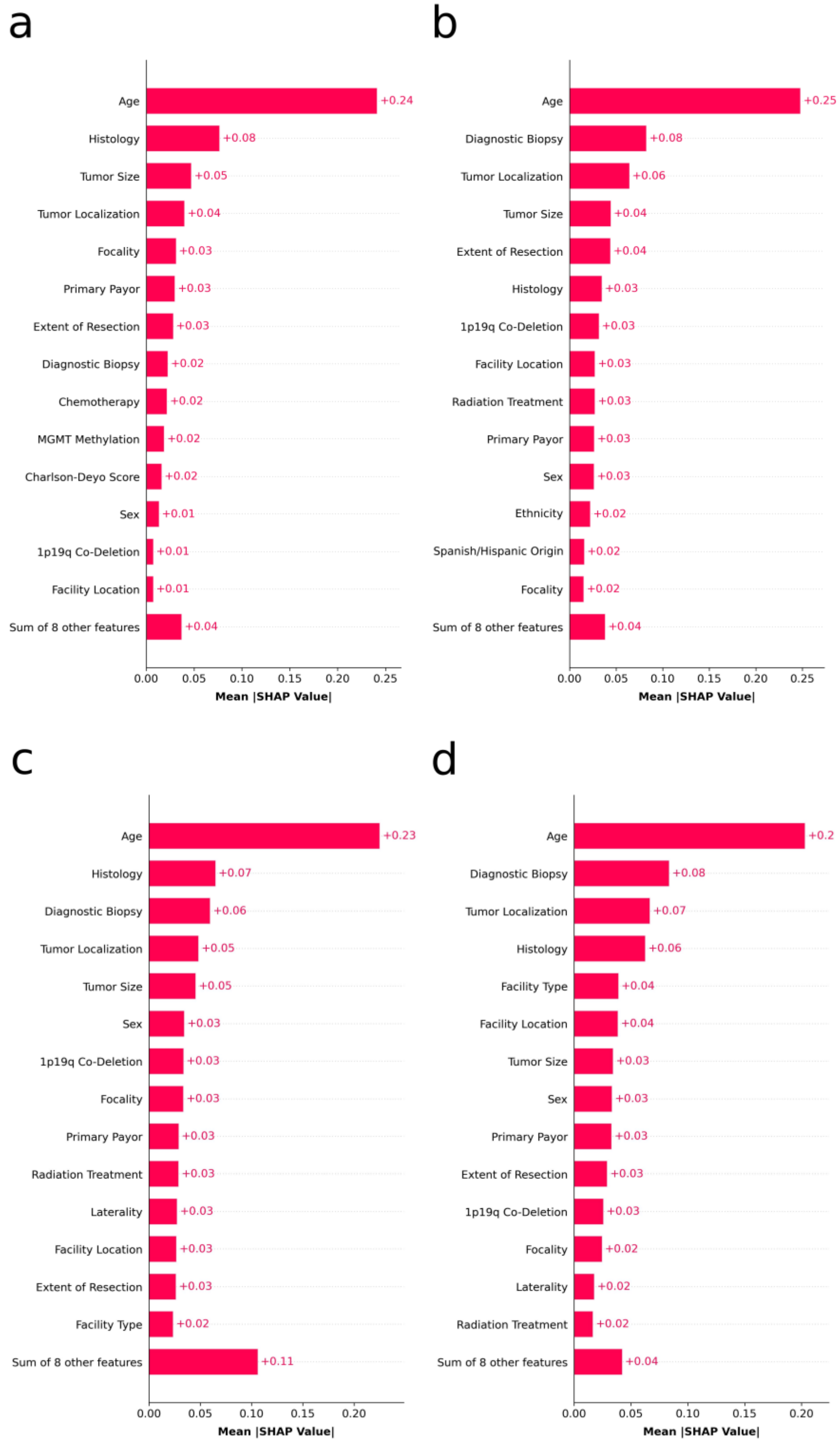

Supplementary Figure 18. The partial dependency plot for the 9 most important features of the model predicting the outcome of 12-month mortality for WHO grade II gliomas with the Random Forest algorithm.

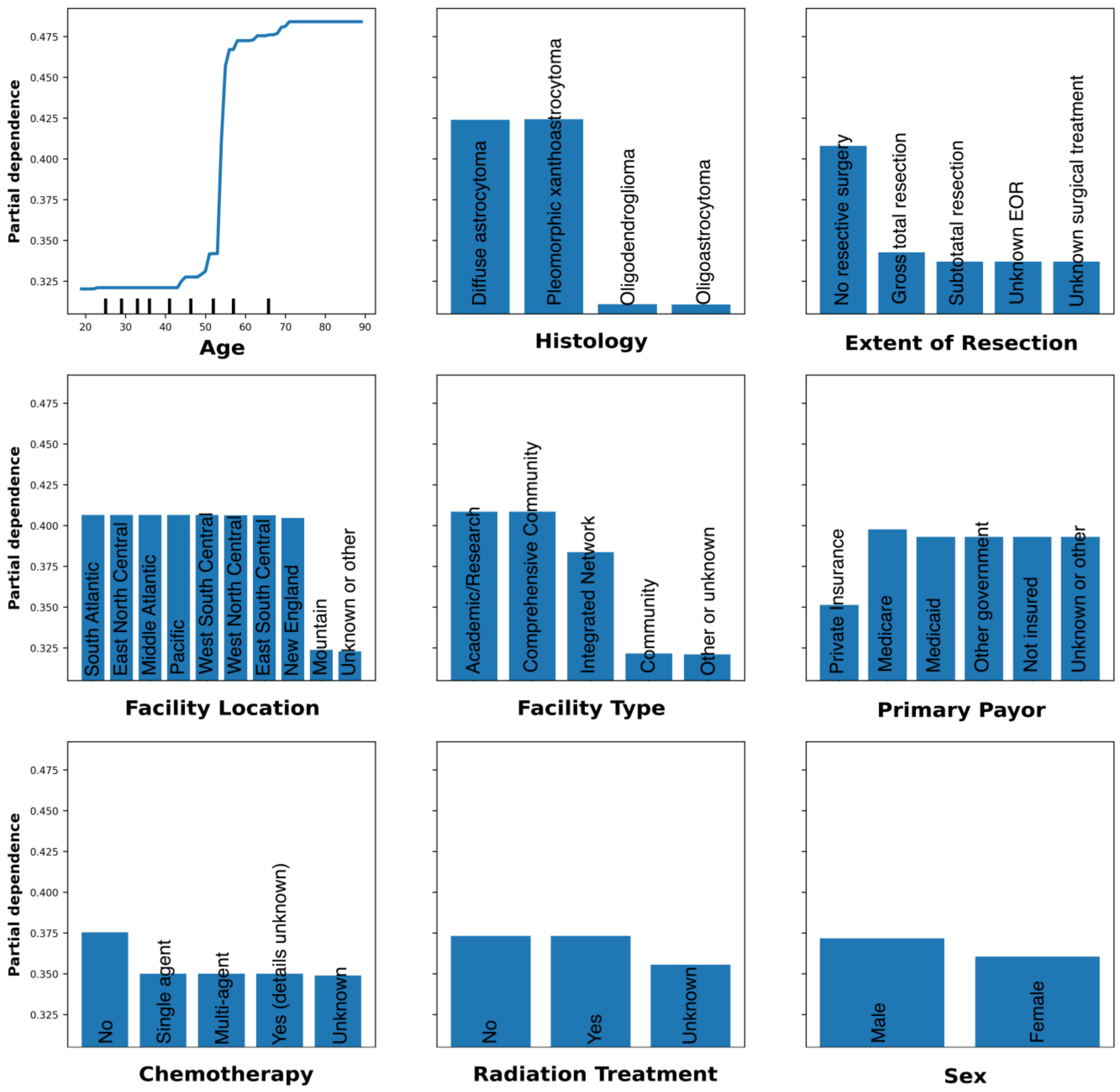

Supplementary Figure 19. The partial dependency plot for the 9 most important features of the model predicting the outcome of 24-month mortality for WHO grade II gliomas with the LightGBM algorithm.

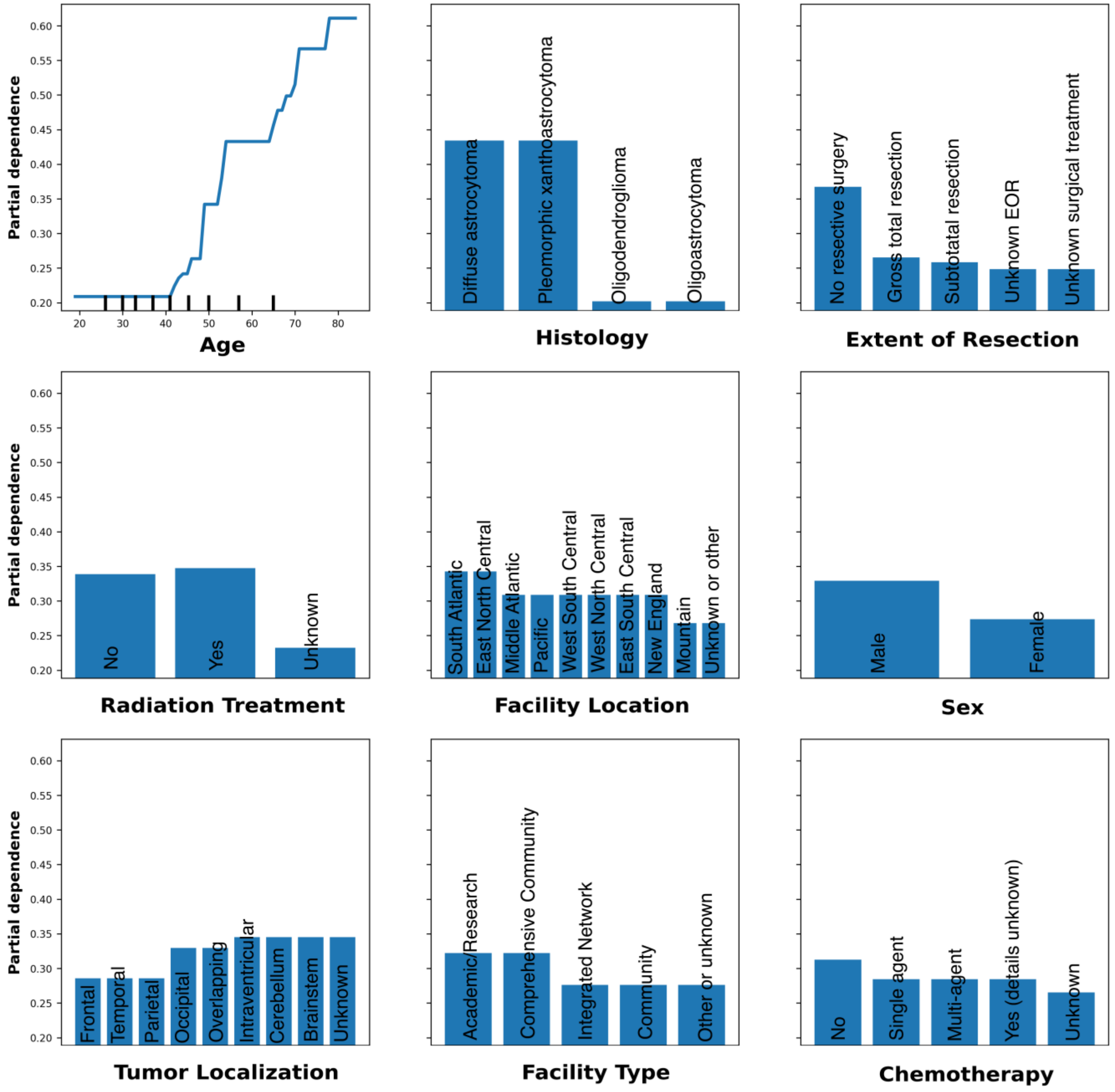

Supplementary Figure 20. The partial dependency plot for the 9 most important features of the model predicting the outcome of 36-month mortality for WHO grade II gliomas with the LightGBM algorithm.

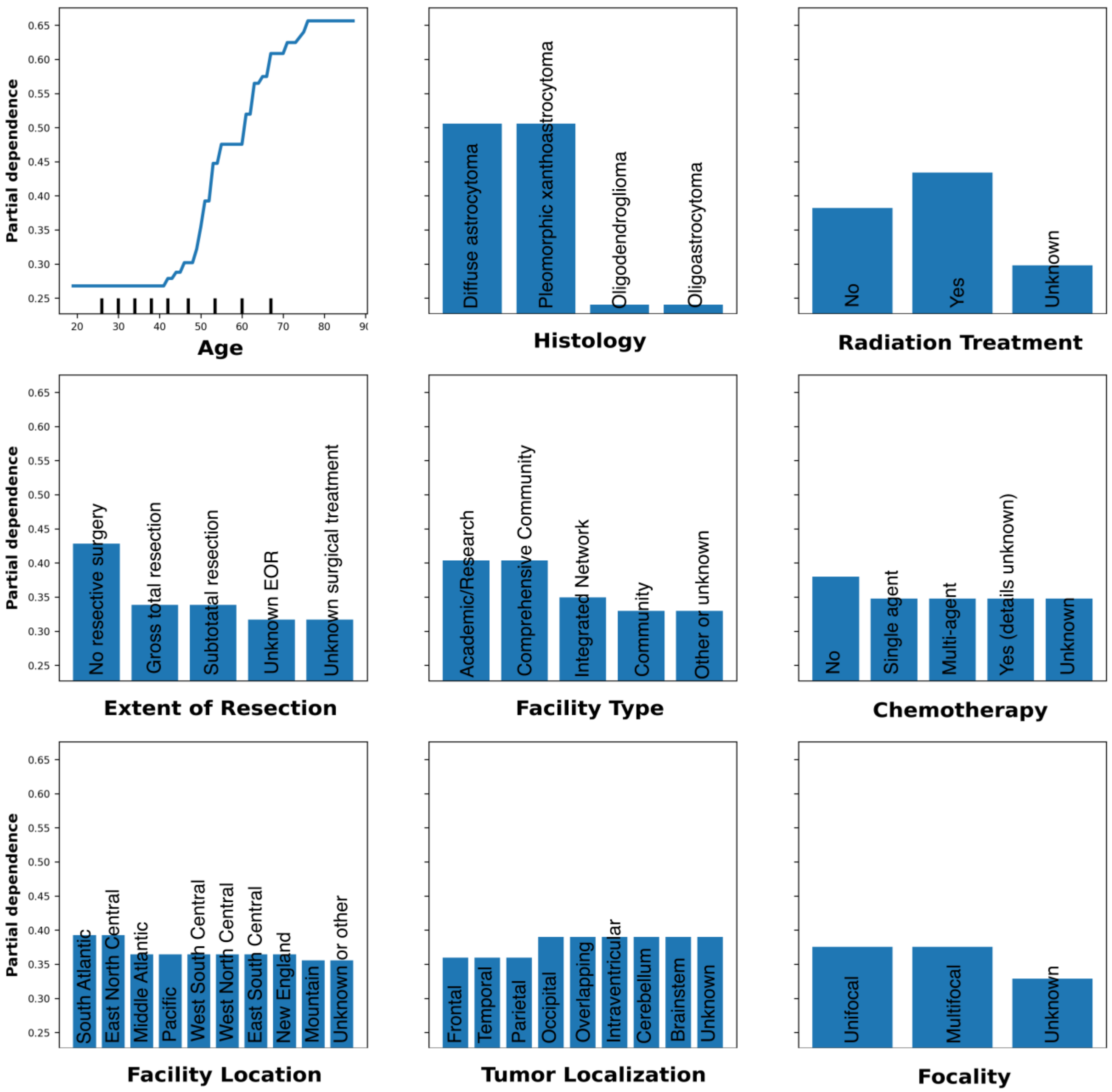

Supplementary Figure 21. The partial dependency plot for the 9 most important features of the model predicting the outcome of 60-month mortality for WHO grade II gliomas with the Random Forest algorithm.

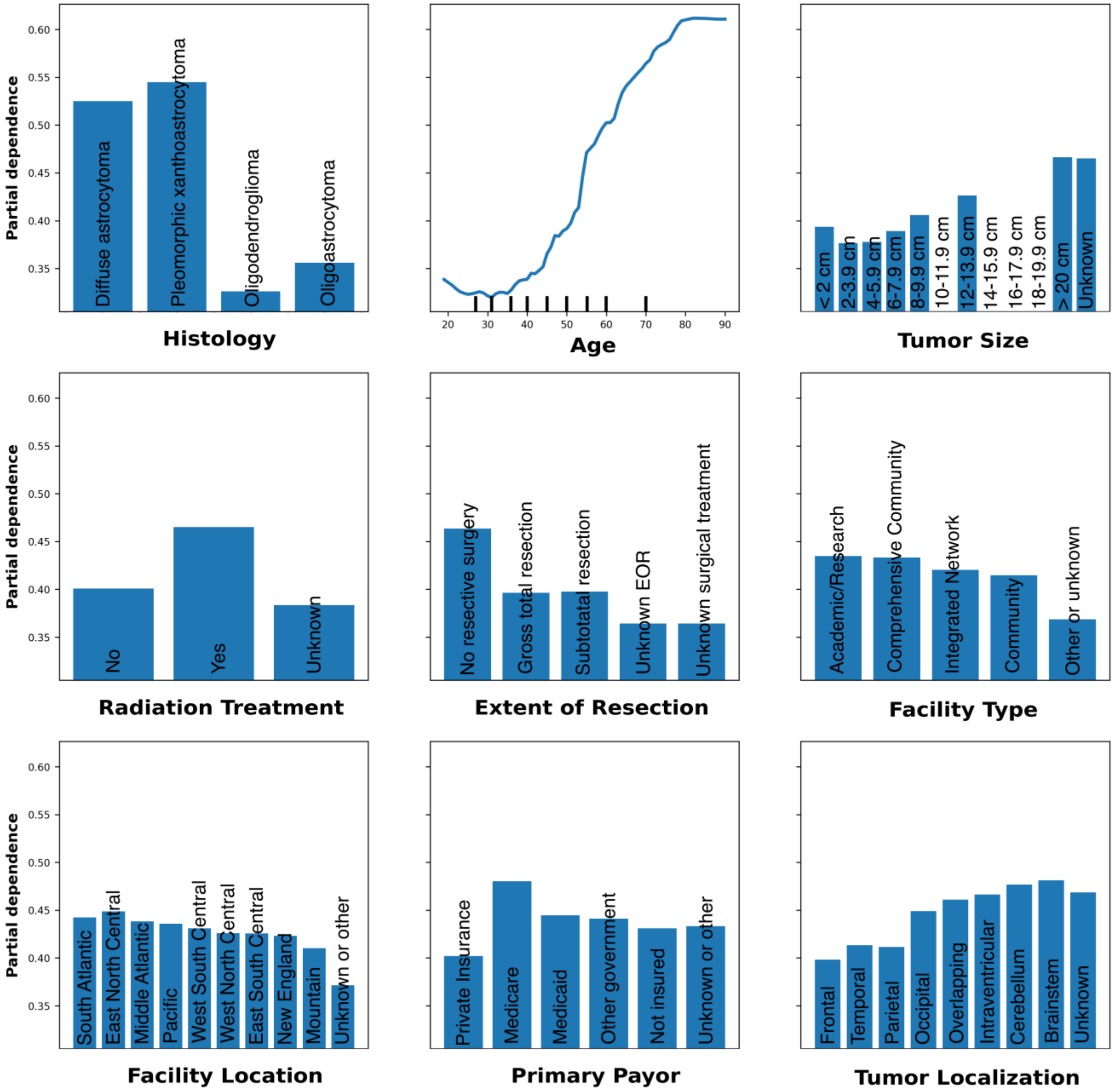

Supplementary Figure 22. The partial dependency plot for the 9 most important features of the model predicting the outcome of 12-month mortality for WHO grade III gliomas with the LightGBM algorithm.

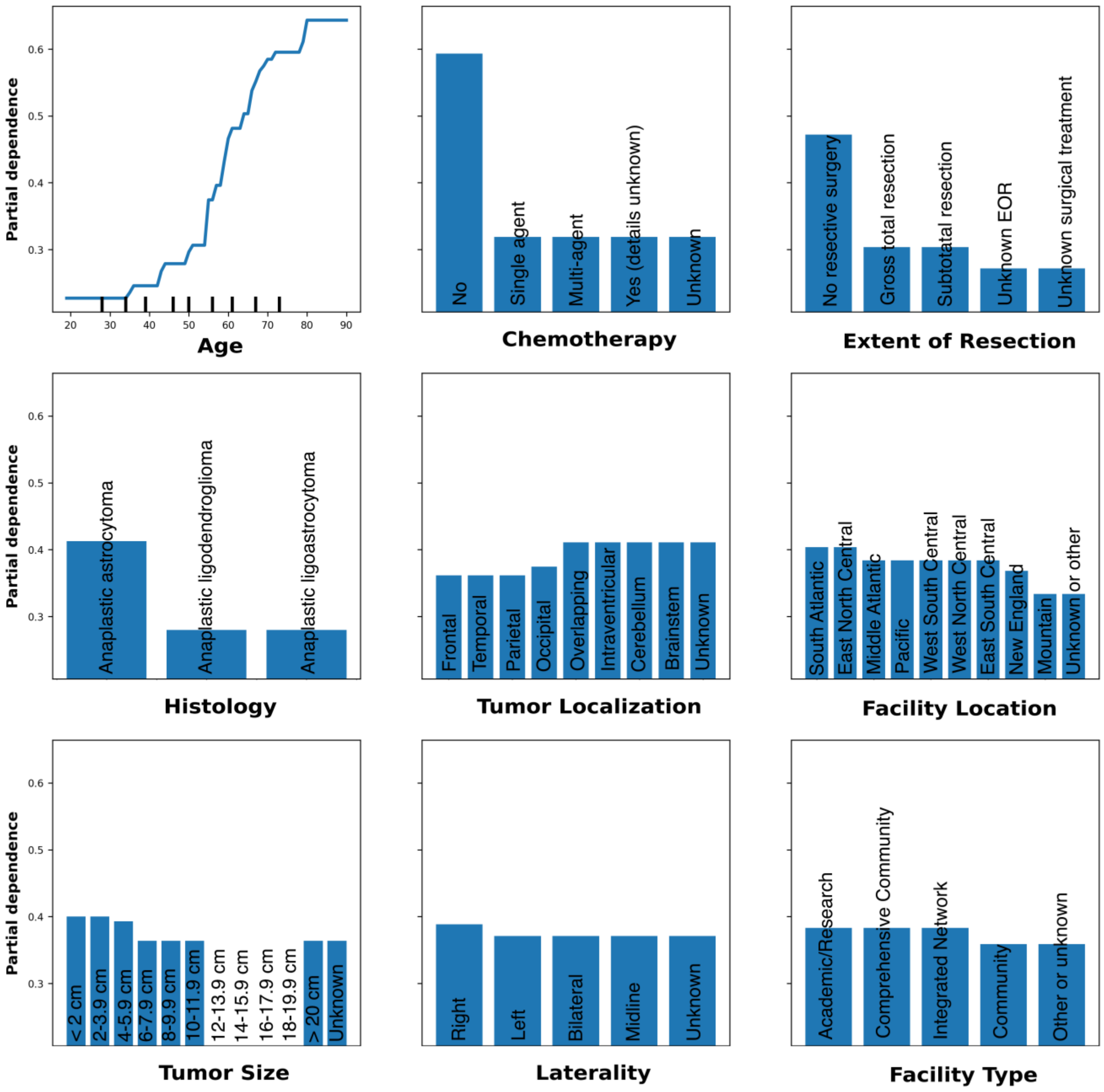

Supplementary Figure 23. The partial dependency plot for the 9 most important features of the model predicting the outcome of 24-month mortality for WHO grade III gliomas with the Random Forest algorithm.

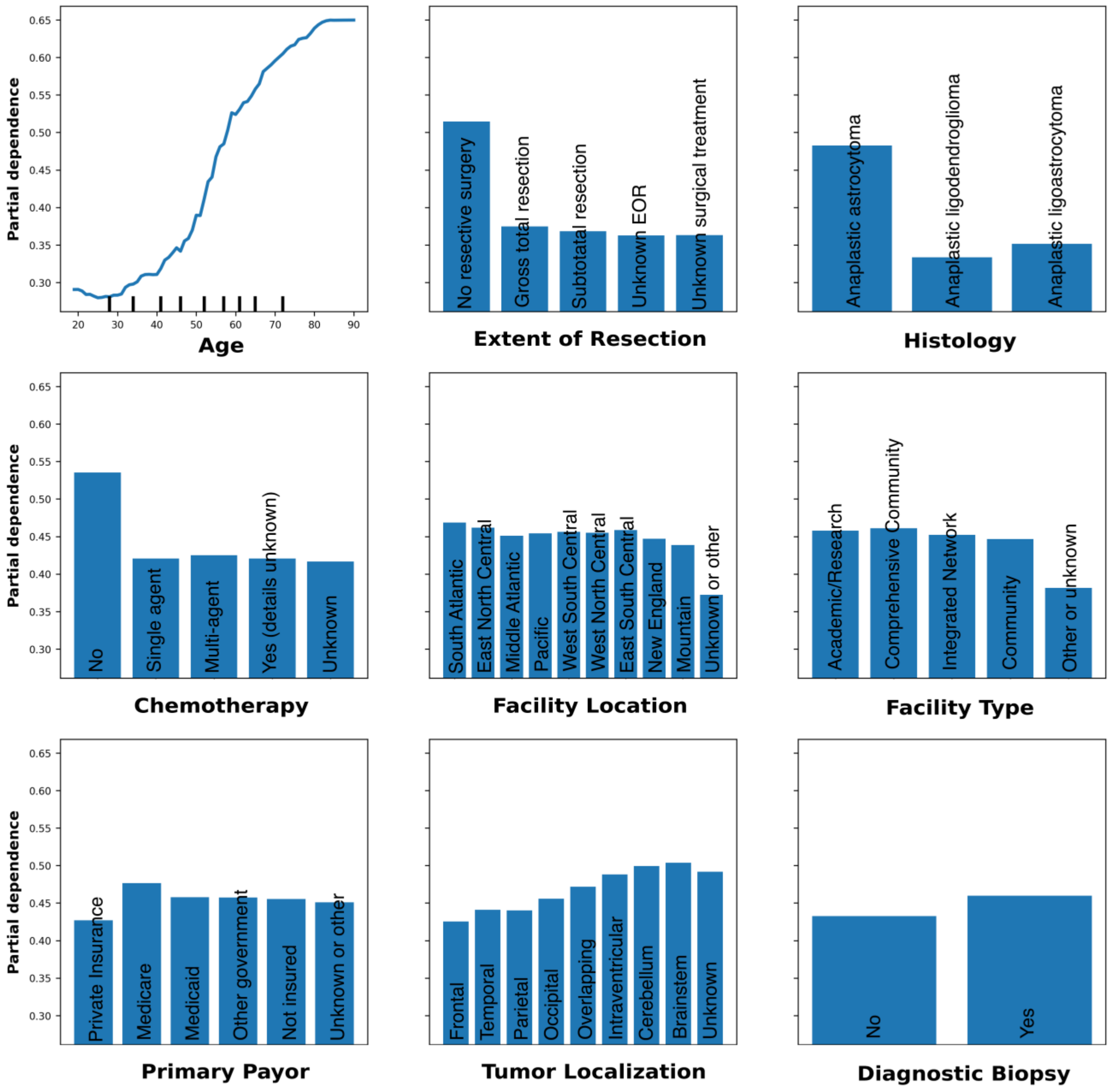

Supplementary Figure 24. The partial dependency plot for the 9 most important features of the model predicting the outcome of 36-month mortality for WHO grade III gliomas with the Random Forest algorithm.

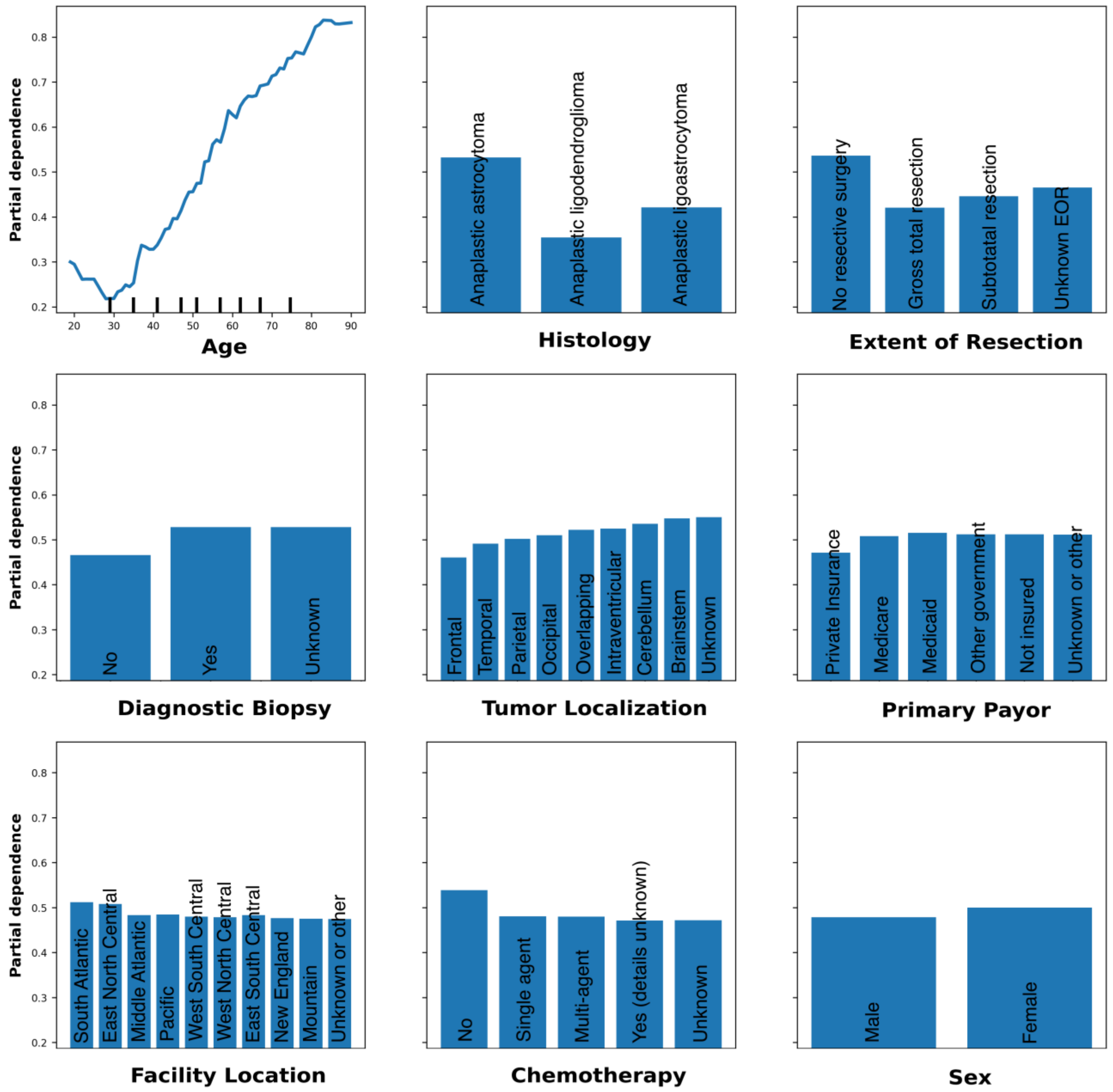

Supplementary Figure 25. The partial dependency plot for the 9 most important features of the model predicting the outcome of 60-month mortality for WHO grade III gliomas with the LightGBM algorithm.

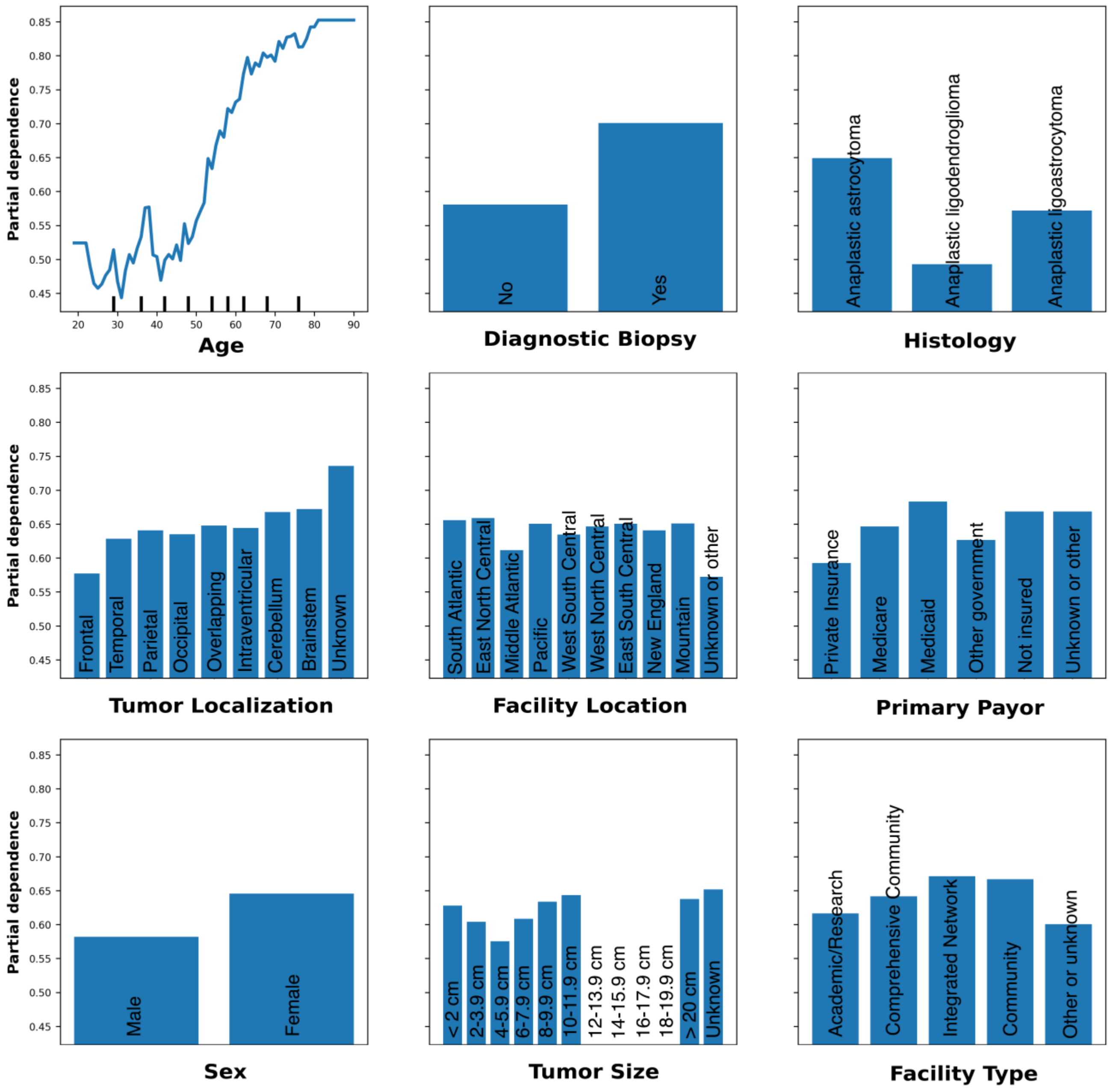

Supplementary Table 1. Detailed patient characteristics. For mortality outcomes, in cases where a patient was logged as alive, but their latest follow-up data ['Last Contact or Death (Months from Diagnosis)'] was recorded prior to the specific survival time point in question, they were omitted from the pertinent survival analyses. The numbers of included patients for each mortality outcome were delineated as follows: 9,748 for 12-month mortality in grade II patients; 11,161 for 12-month mortality in grade III patients; 9,462 for 24-month mortality in grade II patients; 10,943 for 24-month mortality in grade III patients; 8,938 for 36-month mortality in grade II patients; 10,572 for 36-month mortality in grade III patients; 6,763 for 60-month mortality in grade II patients; and 9,095 for 60-month mortality in grade III patients (SD, standard deviation; IQR, interquartile range; n, number; KPS, Karnofsky Performance Scale; MGMT, O6-Methylguanine-DNA Methyltransferase).

**Variables**		**Grade II** (n = 10001)	**Grade III** (n = 11456)	**p Values**	**Total** (n = 21457)
		Mean (±SD), Median (IQR), or n (%)			Mean (±SD), Median (IQR), or n (%)
**Age**		42.0 (23.0)	51.0 (27.0)	<0.001	47.0 (±26.0)
**Sex**	Male	5645 (55.9%)	6357 (55.5%)	0.569	12002 (55.7%)
	Female	4456 (44.1%)	5099 (44.5%)		9555 (44.3%)
**Ethnicity**	White	8889 (88.0%)	10145 (88.6%)	0.037	19034 (88.3%)
	Black	625 (6.2%)	641 (5.6%)		1266 (5.9%)
	Asian Indian or Pakistani	72 (0.7%)	102 (0.9%)		174 (0.8%)
	American Indian, Aleutian, or Eskimo	45 (0.4%)	35 (0.3%)		80 (0.4%)
	Chinese	29 (0.3%)	55 (0.5%)		84 (0.4%)
	Filipino	28 (0.3%)	30 (0.3%)		58 (0.3%)
	Vietnamese	20 (0.2%)	18 (0.2%)		38 (0.2%)
	Korean	10 (0.1%)	19 (0.2%)		29 (0.1%)
	Other or Unknown	383 (3.8%)	411 (3.6%)		794 (3.7%)
**Spanish/Hispanic Origin**	No	9017 (89.3%)	10385 (90.6%)	0.003	19402 (90.0%)
	Yes	782 (7.7%)	782 (6.8%)		1564 (7.3%)
	Unknown	302 (3.0%)	289 (2.5%)		591 (2.7%)
**Primary Payor**	Private insurance	6524 (64.6%)	6582 (57.4%)	<0.001	13106 (60.8%)
	Medicare	1308 (13.0%)	2709 (23.6%)		4017 (18.6%)
	Medicaid	1242 (12.3%)	1242 (10.8%)		2484 (11.5%)
	Not insured	616 (6.1%)	518 (4.5%)		1134 (5.3%)
	Other government	245 (2.4%)	231 (2.0%)		476 (2.2%)
	Unknown	166 (1.6%)	174 (1.5%)		340 (1.6%)

**Facility Type**	Academic/Research Program	3072 (30.4%)	3992 (34.8%)	<0.001	7064 (32.8%)
	Comprehensive Community Cancer Program	1423 (14.1%)	2354 (20.6%)		3777 (17.5%)
	Integrated Network Cancer Program	939 (9.3%)	1622 (14.2%)		2561 (11.9%)
	Community Cancer Program	163 (1.6%)	271 (2.4%)		434 (2.0%)
	Other or Unknown	4504 (44.6%)	3217 (28.1%)		7721 (35.8%)
**Facility Location**	South Atlantic	1128 (11.2%)	1682 (14.7%)	<0.001	2810 (13.0%)
	East North Central	1053 (10.4%)	1361 (11.9%)		2414 (11.2%)
	Middle Atlantic	823 (8.2%)	1354 (11.8%)		2177 (10.1%)
	Pacific	728 (7.2%)	1138 (9.9%)		1866 (8.7%)
	West North Central	474 (4.7%)	777 (6.8%)		1251 (5.8%)
	West South Central	480 (4.8%)	560 (4.9%)		1040 (4.8%)
	East South Central	384 (3.8%)	472 (4.1%)		856 (4.0%)
	Mountain	270 (2.7%)	481 (4.2%)		751 (3.5%)
	New England	257 (2.5%)	414 (3.6%)		671 (3.1%)
	Unknown or Other	4504 (44.6%)	3217 (28.1%)		7721 (35.8%)
**Charlson-Deyo Score**	0	8094 (80.1%)	8891 (77.6%)	<0.001	16985 (78.8%)
	1	1297 (12.8%)	1605 (14.0%)		2902 (13.5%)
	2	453 (4.5%)	602 (5.2%)		1055 (4.9%)
	Greater than 3	257 (2.5%)	358 (3.1%)		615 (2.8%)
**Karnofsky Performance Scale**	KPS 81-100	822 (8.1%)	768 (6.7%)	<0.001	1590 (7.4%)
	KPS 61-80	343 (3.4%)	517 (4.5%)		860 (4.0%)
	KPS 41-60	58 (0.6%)	128 (1.1%)		186 (0.9%)
	KPS 21-40	13 (0.1%)	27 (0.2%)		40 (0.2%)
	KPS 0-20	63 (0.6%)	61 (0.5%)		124 (0.6%)
	Unknown	8802 (87.1%)	9955 (86.9%)		18757 (87.0%)

**Laterality**	Left	4108 (40.7%)	4475 (39.1%)	<0.001	8583 (39.8%)
	Right	3990 (39.5%)	4362 (38.1%)		8352 (38.7%)
	Bilateral	63 (0.6%)	141 (1.2%)		204 (1.0%)
	Midline	28 (0.3%)	37 (0.3%)		65 (0.3%)
	Unknown	1912 (18.9%)	2441 (21.3%)		4353 (20.2%)
**Tumor Localization**	Frontal lobe	4765 (47.2%)	4763 (41.6%)	<0.001	9528 (44.2%)
	Temporal lobe	1940 (19.2%)	2285 (20.0%)		4225 (19.6%)
	Overlapping lesion	945 (9.4%)	1224 (10.7%)		2169 (10.1%)
	Parietal lobe	938 (9.3%)	1157 (10.1%)		2095 (9.7%)
	Occipital lobe	109 (1.1%)	178 (1.6%)		287 (1.3%)
	Brain stem	111 (1.1%)	136 (1.2%)		247 (1.2%)
	Cerebellum	82 (0.8%)	124 (1.1%)		206 (1.0%)
	Intraventricular	40 (0.4%)	40 (0.4%)		80 (0.4%)
	Unknown	1171 (11.6%)	1549 (13.5%)		2720 (12.6%)
**Focality**	Unifocal	7929 (78.5%)	8375 (73.1%)	<0.001	16304 (75.6%)
	Multifocal	374 (3.7%)	998 (8.7%)		1372 (6.4%)
	Unknown	1798 (17.8%)	2083 (18.2%)		3881 (18.0%)
**Diagnostic Biopsy**	No	7181 (71.1%)	7671 (67.0%)	<0.001	14852 (68.9%)
	Yes	2916 (28.9%)	3782 (33.0%)		6698 (31.1%)
	Unknown	4 (0.0%)	3 (0.0%)		7 (0.0%)
**Tumor Size**	< 2 cm	480 (4.8%)	655 (5.7%)	0.015	1135 (5.3%)
	2 - 3.9 cm	1823 (18.0%)	1986 (17.3%)		3809 (17.7%)
	4 - 5.9 cm	1706 (16.9%)	1944 (17.0%)		3650 (16.9%)
	6 - 7.9 cm	922 (9.1%)	1110 (9.7%)		2032 (9.4%)
	8 - 9.9 cm	254 (2.5%)	310 (2.7%)		564 (2.6%)
	10 - 11.9 cm	36 (0.4%)	38 (0.3%)		74 (0.3%)
	12 - 13.9 cm	4 (0.0%)	3 (0.0%)		7 (0.0%)
	14 - 15.9 cm	0 (0.0%)	2 (0.0%)		2 (0.0%)
	16 - 17.9 cm	0 (0.0%)	4 (0.0%)		4 (0.0%)
	> 20 cm	29 (0.3%)	23 (0.2%)		52 (0.2%)
	Unknown	4847 (48.0%)	5381 (47.0%)		10228 (47.4%)

**Histology**	Anaplastic astrocytoma	-	8102 (70.7%)	NA	8102 (37.6%)
	Diffuse astrocytoma	4291 (42.5%)	-		4291 (19.9%)
	Oligodendroglioma	4129 (40.9%)	-		4129 (19.2%)
	Anaplastic oligodendroglioma	-	2138 (18.7%)		2138 (9.9%)
	Oligoastrocytoma	1449 (14.4%)	-		1449 (6.7%)
	Anaplastic oligoastrocytoma	-	1216 (10.6%)		1216 (5.6%)
	Pleomorphic xanthoastrocytoma	232 (2.3%)	-		232 (1.1%)
**1p19q Co-Deletion**	No	1753 (17.4%)	1874 (16.4%)	<0.001	3627 (16.8%)
	Yes	1939 (19.2%)	1244 (10.9%)		3183 (14.8%)
	Unknown	6409 (63.4%)	8338 (72.8%)		14747 (68.4%)
**MGMT Methylation**	Methylated	763 (7.6%)	1139 (9.9%)	<0.001	1902 (8.8%)
	Unmethylated	667 (6.6%)	1084 (9.5%)		1751 (8.1%)
	Unknown	8671 (85.8%)	9233 (80.6%)		17904 (83.0%)
**Ki-67 Labeling Index**	0-20%	3498 (34.6%)	2951 (25.8%)	<0.001	6449 (29.9%)
	21-40%	93 (0.9%)	605 (5.3%)		698 (3.2%)
	41-60%	55 (0.5%)	179 (1.6%)		234 (1.1%)
	61-80%	22 (0.2%)	59 (0.5%)		81 (0.4%)
	81-100%	12 (0.1%)	25 (0.2%)		37 (0.2%)
	Normal (no percentage available)	205 (2.0%)	46 (0.4%)		251 (1.2%)
	Slightly elevated (no percentage available)	204 (2.0%)	135 (1.2%)		339 (1.6%)
	Elevated (no percentage available)	125 (1.2%)	409 (3.6%)		534 (2.5%)
	Unknown	5887 (58.3%)	7047 (61.5%)		12934 (60.0%)

**Extent of Resection**	No resective surgery was performed	3779 (37.4%)	4877 (42.6%)	<0.001	8656 (40.2%)
	Gross total resection	2824 (28.0%)	2765 (24.1%)		5589 (25.9%)
	Subtotal resection	2352 (23.3%)	2616 (22.8%)		4968 (23.0%)
	Resective surgery was performed but the extent of resection is unknown	1130 (11.2%)	1182 (10.3%)		2312 (10.7%)
	Unknown whether resective surgery is performed or not	16 (0.2%)	16 (0.1%)		32 (0.2%)
**Radiation Treatment**	Yes	3974 (39.3%)	8694 (75.9%)	<0.001	12668 (58.8%)
	No	1750 (17.3%)	541 (4.7%)		2291 (10.6%)
	Unknown	4377 (43.3%)	2221 (19.4%)		6598 (30.6%)
**Chemotherapy**	Yes(single-agent chemotherapy)	3138 (31.1%)	7727 (67.4%)	<0.001	10865 (50.4%)
	Yes (multiagent chemotherapy)	378 (3.7%)	426 (3.7%)		804 (3.7%)
	Yes (details unknown)	173 (1.7%)	296 (2.6%)		469 (2.2%)
	No	6038 (59.8%)	2619 (22.9%)		8657 (40.2%)
	Unknown	374 (3.7%)	388 (3.4%)		762 (3.5%)
**Immunotherapy**	No	10044 (99.4%)	11252 (98.2%)	<0.001	21296 (98.8%)
	Yes	36 (0.4%)	177 (1.6%)		213 (1.0%)
	Unknown	21 (0.2%)	27 (0.2%)		48 (0.2%)
**12-Month Mortality***	Yes	655 (6.7%)	2841 (25.5%)	<0.001	3496 (16.7%)
	No	9093 (93.3%)	8320 (74.5%)		17413 (83.3%)
**24-Month Mortality***	Yes	1202 (12.7%)	4493 (41.1%)	<0.001	5695 (27.9%)
	No	8260 (87.3%)	6450 (58.9%)		14710 (72.1%)
**36-Month Mortality***	Yes	1597 (17.9%)	5389 (51%)	<0.001	6984 (35.9%)
	No	7341 (83.1%)	5183 (49%)		12524 (64.1%)
**60-Month Mortality***	Yes	2225 (32.9%)	6141 (67.5%)	<0.001	8366 (52.8%)
	No	4538 (67.1%)	2954 (32.5%)		7492 (57.2%)

Supplementary Table 2. Performance metrics of the models (CI, confidence interval; AUPRC, area under the precision-recall curve; AUROC, area under the receiver operating characteristics curve).

	Outcome	Algorithm	Sensitivity (95% CI)	Specificity (95% CI)	Accuracy (95% CI)	AUPRC (95% CI)	AUROC (95% CI)	Brier Score (95% CI)
Grade II	12-Month Mortality	TabPFN	0.0 (0.0 - 0.0)	1.0 (1.0 - 1.0)	0.927 (0.915 - 0.939)	0.454 (0.432 - 0.476)	0.879 (0.864 - 0.915)	0.054 (0.044 - 0.064)
		TabNet	0.556 (0.534 - 0.578)	0.89 (0.876 - 0.904)	0.866 (0.851 - 0.881)	0.288 (0.268 - 0.308)	0.814 (0.769 - 0.849)	0.06 (0.049 - 0.071)
		XGBoost	0.711 (0.691 - 0.731)	0.869 (0.854 - 0.884)	0.858 (0.843 - 0.873)	0.37 (0.349 - 0.391)	0.862 (0.851 - 0.904)	0.054 (0.044 - 0.064)
		LightGBM	0.662 (0.641 - 0.683)	0.892 (0.878 - 0.906)	0.875 (0.86 - 0.89)	0.362 (0.341 - 0.383)	0.871 (0.824 - 0.889)	0.055 (0.045 - 0.065)
		**Random Forest**	**0.838 (0.822 - 0.854)**	**0.814 (0.797 - 0.831)**	**0.816 (0.799 - 0.833)**	**0.383 (0.361 - 0.405)**	**0.888 (0.856 - 0.912)**	**0.054 (0.044 - 0.064)**
	24-Month Mortality	TabPFN	0.239 (0.22 - 0.258)	0.979 (0.973 - 0.985)	0.884 (0.87 - 0.898)	0.512 (0.489 - 0.535)	0.854 (0.816 - 0.871)	0.086 (0.073 - 0.099)
		TabNet	0.617 (0.595 - 0.639)	0.844 (0.828 - 0.86)	0.815 (0.798 - 0.832)	0.437 (0.415 - 0.459)	0.8 (0.768 - 0.83)	0.09 (0.077 - 0.103)
		XGBoost	0.667 (0.646 - 0.688)	0.819 (0.802 - 0.836)	0.8 (0.782 - 0.818)	0.388 (0.366 - 0.41)	0.83 (0.787 - 0.845)	0.112 (0.098 - 0.126)
		**LightGBM**	**0.712 (0.692 - 0.732)**	**0.839 (0.822 - 0.856)**	**0.823 (0.806 - 0.84)**	**0.523 (0.5 - 0.546)**	**0.859 (0.804 - 0.867)**	**0.083 (0.071 - 0.095)**
		Random Forest	0.728 (0.708 - 0.748)	0.799 (0.781 - 0.817)	0.79 (0.772 - 0.808)	0.496 (0.473 - 0.519)	0.826 (0.81 - 0.867)	0.085 (0.072 - 0.098)

	**36-Month Mortality**	TabPFN	0.362<br>(0.34 - 0.384)	0.957<br>(0.948 - 0.966)	0.85<br>(0.833 - 0.867)	0.553<br>(0.53 - 0.576)	0.799<br>(0.782 - 0.838)	0.111<br>(0.096 - 0.126)
		TabNet	0.613<br>(0.59 - 0.636)	0.823<br>(0.805 - 0.841)	0.785<br>(0.766 - 0.804)	0.51<br>(0.487 - 0.533)	0.807<br>(0.771 - 0.829)	0.114<br>(0.099 - 0.129)
		XGBoost	0.591<br>(0.568 - 0.614)	0.863<br>(0.847 - 0.879)	0.814<br>(0.796 - 0.832)	0.547<br>(0.524 - 0.57)	0.796<br>(0.764 - 0.824)	0.114<br>(0.099 - 0.129)
		**LightGBM**	**0.653<br>(0.631 - 0.675)**	**0.836<br>(0.819 - 0.853)**	**0.803<br>(0.785 - 0.821)**	**0.564<br>(0.541 - 0.587)**	**0.813<br>(0.777 - 0.835)**	**0.111<br>(0.096 - 0.126)**
		Random Forest	0.632<br>(0.61 - 0.654)	0.841<br>(0.824 - 0.858)	0.803<br>(0.785 - 0.821)	0.547<br>(0.524 - 0.57)	0.795<br>(0.783 - 0.839)	0.111<br>(0.096 - 0.126)
	**60-Month Mortality**	TabPFN	0.578<br>(0.552 - 0.604)	0.906<br>(0.89 - 0.922)	0.801<br>(0.78 - 0.822)	0.738<br>(0.715 - 0.761)	0.828<br>(0.802 - 0.85)	0.149<br>(0.13 - 0.168)
		TabNet	0.698<br>(0.674 - 0.722)	0.797<br>(0.776 - 0.818)	0.766<br>(0.743 - 0.789)	0.706<br>(0.682 - 0.73)	0.808<br>(0.777 - 0.83)	0.155<br>(0.136 - 0.174)
		XGBoost	0.654<br>(0.629 - 0.679)	0.826<br>(0.806 - 0.846)	0.772<br>(0.75 - 0.794)	0.718<br>(0.694 - 0.742)	0.834<br>(0.795 - 0.845)	0.151<br>(0.132 - 0.17)
		LightGBM	0.731<br>(0.707 - 0.755)	0.805<br>(0.784 - 0.826)	0.781<br>(0.759 - 0.803)	0.75<br>(0.727 - 0.773)	0.825<br>(0.814 - 0.86)	0.143<br>(0.124 - 0.162)
		**Random Forest**	**0.684<br>(0.659 - 0.709)**	**0.835<br>(0.815 - 0.855)**	**0.787<br>(0.765 - 0.809)**	**0.748<br>(0.725 - 0.771)**	**0.846<br>(0.815 - 0.863)**	**0.142<br>(0.123 - 0.161)**

**Grade III**	**12-Month Mortality**	TabPFN	0.539 (0.518 - 0.56)	0.924 (0.913 - 0.935)	0.826 (0.81 - 0.842)	0.704 (0.685 - 0.723)	0.872 (0.851 - 0.884)	0.125 (0.111 - 0.139)
		TabNet	0.676 (0.657 - 0.695)	0.807 (0.791 - 0.823)	0.773 (0.756 - 0.79)	0.611 (0.591 - 0.631)	0.811 (0.801 - 0.839)	0.143 (0.128 - 0.158)
		XGBoost	0.71 (0.691 - 0.729)	0.853 (0.838 - 0.868)	0.817 (0.801 - 0.833)	0.717 (0.698 - 0.736)	0.86 (0.856 - 0.889)	0.119 (0.106 - 0.132)
		**LightGBM**	**0.768 (0.75 - 0.786)**	**0.811 (0.795 - 0.827)**	**0.8 (0.783 - 0.817)**	**0.725 (0.706 - 0.744)**	**0.876 (0.857 - 0.889)**	**0.119 (0.106 - 0.132)**
		Random Forest	0.72 (0.701 - 0.739)	0.847 (0.832 - 0.862)	0.815 (0.799 - 0.831)	0.724 (0.705 - 0.743)	0.871 (0.862 - 0.892)	0.118 (0.105 - 0.131)
	**24-Month Mortality**	TabPFN	0.64 (0.62 - 0.66)	0.86 (0.845 - 0.875)	0.785 (0.768 - 0.802)	0.772 (0.754 - 0.79)	0.837 (0.823 - 0.858)	0.161 (0.146 - 0.176)
		TabNet	0.688 (0.669 - 0.707)	0.798 (0.781 - 0.815)	0.748 (0.73 - 0.766)	0.754 (0.736 - 0.772)	0.828 (0.808 - 0.843)	0.166 (0.15 - 0.182)
		XGBoost	0.73 (0.711 - 0.749)	0.799 (0.782 - 0.816)	0.784 (0.767 - 0.801)	0.771 (0.753 - 0.789)	0.841 (0.825 - 0.858)	0.159 (0.144 - 0.174)
		LightGBM	0.738 (0.72 - 0.756)	0.8 (0.783 - 0.817)	0.794 (0.777 - 0.811)	0.775 (0.758 - 0.792)	0.853 (0.837 - 0.869)	0.155 (0.14 - 0.17)
		**Random Forest**	**0.722 (0.703 - 0.741)**	**0.81 (0.794 - 0.826)**	**0.796 (0.779 - 0.813)**	**0.775 (0.758 - 0.792)**	**0.855 (0.839 - 0.87)**	**0.153 (0.138 - 0.168)**

	**36-Month Mortality**	TabPFN	0.713 (0.694 - 0.732)	0.831 (0.815 - 0.847)	0.865 (0.85 - 0.88)	0.771 (0.753 - 0.789)	0.853 (0.842 - 0.872)	0.155 (0.14 - 0.17)
		TabNet	0.723 (0.704 - 0.742)	0.833 (0.817 - 0.849)	0.857 (0.842 - 0.872)	0.777 (0.759 - 0.795)	0.845 (0.838 - 0.871)	0.154 (0.139 - 0.169)
		XGBoost	0.77 (0.752 - 0.788)	0.804 (0.787 - 0.821)	0.857 (0.842 - 0.872)	0.786 (0.769 - 0.803)	0.854 (0.842 - 0.874)	0.153 (0.138 - 0.168)
		LightGBM	0.777 (0.759 - 0.795)	0.833 (0.817 - 0.849)	0.882 (0.868 - 0.896)	0.804 (0.787 - 0.821)	0.856 (0.861 - 0.89)	0.143 (0.128 - 0.158)
		**Random Forest**	**0.763 (0.745 - 0.781)**	**0.827 (0.811 - 0.843)**	**0.874 (0.86 - 0.888)**	**0.794 (0.777 - 0.811)**	**0.878 (0.857 - 0.885)**	**0.146 (0.131 - 0.161)**
	**60-Month Mortality**	TabPFN	0.845 (0.828 - 0.862)	0.652 (0.63 - 0.674)	0.924 (0.912 - 0.936)	0.786 (0.767 - 0.805)	0.852 (0.825 - 0.863)	0.147 (0.131 - 0.163)
		TabNet	0.775 (0.756 - 0.794)	0.72 (0.699 - 0.741)	0.905 (0.892 - 0.918)	0.758 (0.738 - 0.778)	0.826 (0.798 - 0.84)	0.157 (0.14 - 0.174)
		XGBoost	0.82 (0.802 - 0.838)	0.716 (0.695 - 0.737)	0.924 (0.912 - 0.936)	0.788 (0.769 - 0.807)	0.84 (0.827 - 0.863)	0.146 (0.13 - 0.162)
		**LightGBM**	**0.816 (0.798 - 0.834)**	**0.748 (0.728 - 0.768)**	**0.93 (0.918 - 0.942)**	**0.795 (0.776 - 0.814)**	**0.86 (0.834 - 0.87)**	**0.142 (0.126 - 0.158)**
		Random Forest	0.803 (0.785 - 0.821)	0.768 (0.749 - 0.787)	0.93 (0.918 - 0.942)	0.792 (0.773 - 0.811)	0.855 (0.834 - 0.87)	0.142 (0.126 - 0.158)

Supplementary Table 3. Hyperparameter spaces.

Algorithm	Hyperparameter	Value Range
**TabNet**	seed	31
	n_d	8 to 64
	n_a	6 to 64
	n_steps	1 to 10
	gamma	1 to 2
	n_independent	1 to 5
	n_shared	1 to 5
	lambda_sparse	1e-6 to 1e-3 (log scale)
	lr	1e-5 to 1e-2 (log scale)
**XGBoost**	seed	31
	verbosity	0
	objective	binary:logistic
	eval_metric	auc
	booster	gbtree
	lambda	1e-8 to 1 (log scale)
	alpha	1e-8 to 1 (log scale)
	max_depth	1 to 9
	eta	1e-8 to 1 (log scale)
	gamma	1e-8 to 1 (log scale)
	grow_policy	depthwise, lossguide
**LightGBM**	objective	binary
	metric	binary_log
	verbosity	-1
	random_state	31
	boosting_type	gbdt
	lambda_l1	1e-8 to 10 (log scale)
	lambda_l2	1e-8 to 10 (log scale)
	num_leaves	2 to 256
	feature_fraction	0.4 to 1.0
	bagging_fraction	0.4 to 1.0
	bagging_freq	1 to 7
	min_child_samples	5 to 100
**Random Forest**	random_state	31
	criterion	gini, entropy
	max_features	auto, sqrt, log2, none
	max_depth	1 to 100
	n_estimators	100 to 2000 (in steps of 100)
	min_samples_leaf	1 to 4
	min_samples_split	2 to 10

Supplementary Table 4. Final hyperparameters.

	Outcome	Algorithm	Hyperparameter	Hyperparameter Value
Grade II	12-Month Mortality	Random Forest	random_state	31
			criterion	gini
			max_features	log2
			max_depth	4
			n_estimators	900
			min_samples_leaf	3
			min_samples_split	3
	24-Month Mortality	LightGBM	objective	binary
			metric	binary_logloss
			verbosity	-1
			random_state	31
			boosting_type	gbdt
			lambda_l1	6.12E-07
			lambda_l2	0.002899842
			num_leaves	2
			feature_fraction	0.679810766
			bagging_fraction	0.429501253
			bagging_freq	7
			min_child_samples	87
	36-Month Mortality	LightGBM	objective	binary
			metric	binary_logloss
			verbosity	-1
			random_state	31
			boosting_type	gbdt
			lambda_l1	6.81E-07
			lambda_l2	0.003064769
			num_leaves	2
			feature_fraction	0.678093183
			bagging_fraction	0.504615021
			bagging_freq	3
			min_child_samples	69
	60-Month Mortality	Random Forest	random_state	31
			criterion	gini
			max_features	log2
			max_depth	5
			n_estimators	800
			min_samples_leaf	3
			min_samples_split	2

Grade III	12-Month Mortality	LightGBM	objective	binary
			metric	binary_logloss
			verbosity	-1
			random_state	31
			boosting_type	gbdt
			lambda_l1	2.96E-04
			lambda_l2	0.000391428
			num_leaves	13
			feature_fraction	0.459866969
			bagging_fraction	0.52938131
			bagging_freq	3
			min_child_samples	93
	24-Month Mortality	Random Forest	random_state	31
			criterion	entropy
			max_features	-
			max_depth	53
			n_estimators	800
			min_samples_leaf	4
			min_samples_split	8
	36-Month Mortality	Random Forest	random_state	31
			criterion	entropy
			max_features	-
			max_depth	44
			n_estimators	1000
			min_samples_leaf	2
			min_samples_split	7
	60-Month Mortality	LightGBM	objective	binary
			metric	binary_logloss
			verbosity	-1
			random_state	31
			boosting_type	gbdt
			lambda_l1	1.97E-06
			lambda_l2	0.001274129
			num_leaves	17
			feature_fraction	0.40138612
			bagging_fraction	0.498393004
			bagging_freq	4
			min_child_samples	91

Supplementary Video 1. Demonstration of the web application.
