# Supplementary material for: Prognosis Individualized: Survival predictions for WHO grade II and III gliomas with a machine learning-based web application
Source: NPJ Digit Med. 2023 Oct 26;6:200. doi: 10.1038/s41746-023-00948-y (PMC10603035; doi:10.1038/s41746-023-00948-y)
Supplement: Supplementary file 3 — Reporting Summary [file 41746_2023_948_MOESM3_ESM.pdf]

nature portfolio

Corresponding author(s): NPJDIGITALMED-07828R

Last updated by author(s): 10/03/2023

Reporting Summary

Nature Portfolio wishes to improve the reproducibility of the work that we publish. This form provides structure for consistency and transparency in reporting. For further information on Nature Portfolio policies, see our Editorial Policies and the Editorial Policy Checklist.

Statistics

For all statistical analyses, confirm that the following items are present in the figure legend, table legend, main text, or Methods section.

n/a	Confirmed	
☐	☒	The exact sample size (*n*) for each experimental group/condition, given as a discrete number and unit of measurement
☒	☐	A statement on whether measurements were taken from distinct samples or whether the same sample was measured repeatedly
☐	☒	The statistical test(s) used AND whether they are one- or two-sided<br>*Only common tests should be described solely by name; describe more complex techniques in the Methods section.*
☐	☒	A description of all covariates tested
☐	☒	A description of any assumptions or corrections, such as tests of normality and adjustment for multiple comparisons
☐	☒	A full description of the statistical parameters including central tendency (e.g. means) or other basic estimates (e.g. regression coefficient) AND variation (e.g. standard deviation) or associated estimates of uncertainty (e.g. confidence intervals)
☐	☒	For null hypothesis testing, the test statistic (e.g. *F*, *t*, *r*) with confidence intervals, effect sizes, degrees of freedom and *P* value noted<br>*Give P values as exact values whenever suitable.*
☒	☐	For Bayesian analysis, information on the choice of priors and Markov chain Monte Carlo settings
☒	☐	For hierarchical and complex designs, identification of the appropriate level for tests and full reporting of outcomes
☒	☐	Estimates of effect sizes (e.g. Cohen's *d*, Pearson's *r*), indicating how they were calculated

Our web collection on statistics for biologists contains articles on many of the points above.

Software and code

Policy information about availability of computer code

Data collection	N/A
Data analysis	The source code for preprocessing and analyzing the data is available on GitHub (https://github.com/mertkarabacak/NCDB-G2G3_Glioma).

For manuscripts utilizing custom algorithms or software that are central to the research but not yet described in published literature, software must be made available to editors and reviewers. We strongly encourage code deposition in a community repository (e.g. GitHub). See the Nature Portfolio guidelines for submitting code & software for further information.

Data

Policy information about availability of data

All manuscripts must include a data availability statement. This statement should provide the following information, where applicable:

- Accession codes, unique identifiers, or web links for publicly available datasets
- A description of any restrictions on data availability
- For clinical datasets or third party data, please ensure that the statement adheres to our policy

Restrictions apply to the availability of the data. Data were obtained from the NCDB, a prospectively maintained repository collaboratively developed by the CoC of the American College of Surgeons and the American Cancer Society. None of these institutions have verified and are not responsible for the statistical validity of the data analysis or the conclusions derived by the authors.

Research involving human participants, their data, or biological material

Policy information about studies with human participants or human data. See also policy information about sex, gender (identity/presentation), and sexual orientation and race, ethnicity and racism.

Reporting on sex and gender	The 'Sex' variable in our study was collected by the National Cancer Database as NAACCR item #220. The National Cancer Database reports that the patient's sex was collected as indicated in the medical record. In the data dictionary (https://www.facs.org/media/brilfbgu/puf-2020-data-dictionary.pdf) it was noted that due to low case counts, any sex other than male or female is suppressed in the participant user files.
Reporting on race, ethnicity, or other socially relevant groupings	The 'Ethnicity' and 'Spanish/Hispanic Origin' variables were collected by the National Cancer Database as NAACCR items #160 and #190. We renamed the collected 'Race' variable as 'Ethnicity' per journal guidelines recommended.
Population characteristics	Our study employed a range of predictor variables that span sociodemographic, clinicopathologic, and treatment-related attributes: 1) sociodemographics: age, sex, ethnicity, Spanish/Hispanic origin, primary payor, facility type, and facility location; 2) clinical presentation: Charlson-Deyo Score (as a measure of comorbidities), and Karnofsky Performance Scale; 3) diagnostic information: diagnostic biopsy (whether a diagnostic biopsy was taken before a possible resective surgery), tumor laterality, localization, focality (unifocal or multifocal), size (as ordinal), and histology; 4) molecular markers: 1p19q co-deletion, MGMT methylation, and Ki-67 labeling index; and 5) treatment modalities: resective surgery, extent of resection, radiation treatment, chemotherapy and immunotherapy. Ethnicity and Spanish/Hispanic origin variables were collected and reported by the NCDB, along with other variables. For detailed information regarding the data items a data dictionary can be found at https://www.facs.org/media/brilfbgu/puf-2020-data-dictionary.pdf.
Recruitment	The NCDB-Brain Participant User File (PUF) was filtered for adults at least 18 years of age diagnosed with histologically confirmed cranial WHO grade II and III gliomas between 2010 and 2017. The initial temporal boundary of 2010 was chosen to reflect the advancements made in the treatment of gliomas over the preceding decade, whereas the terminal boundary of 2017 was selected to confine the study population. This latter limitation served to minimize the exclusion of patients due to the lack of extensive follow-up data. We used the International Classification of Disease for Oncology, third edition (ICD-O-3) histologic codes for diffuse astrocytoma [9400 (grade II)], anaplastic astrocytoma [9401 (grade III)], pleomorphic xanthoastrocytoma [9424 (grade II)], pilomyxoid astrocytoma [9425 (grade II)], oligodendroglioma [9450 (grade II)], anaplastic oligodendroglioma [9451 (grade III)], oligoastrocytoma [9382 (grade II)], and anaplastic oligoastrocytoma [9382 grade III)]; and ICD-O-3 topographical codes C71.0–C71.9 to define our patient population.
Ethics oversight	No institutional review board (IRB) approval or informed consent was required due to the use of de-identified patient data. The study was deemed exempt by the Icahn School of Medicine at Mount Sinai's IRB.

Note that full information on the approval of the study protocol must also be provided in the manuscript.

Field-specific reporting

Please select the one below that is the best fit for your research. If you are not sure, read the appropriate sections before making your selection.

☒ Life sciences ☐ Behavioural & social sciences ☐ Ecological, evolutionary & environmental sciences

For a reference copy of the document with all sections, see nature.com/documents/nr-reporting-summary-flat.pdf

Life sciences study design

All studies must disclose on these points even when the disclosure is negative.

Sample size	No sample size calculation was performed. The NCDB-Brain Participant User File (PUF) was filtered for adults at least 18 years of age diagnosed with histologically confirmed cranial WHO grade II and III gliomas between 2010 and 2017.
Data exclusions	The initial temporal boundary of 2010 was chosen to reflect the advancements made in the treatment of gliomas over the preceding decade, whereas the terminal boundary of 2017 was selected to confine the study population. This latter limitation served to minimize the exclusion of patients due to the lack of extensive follow-up data. Patients with missing data in the 'Vital Status' and 'Last Contact or Death (Months from Diagnosis)' data items were excluded. Moreover, in cases where a patient was logged as alive, but their latest follow-up data ['Last Contact or Death (Months from Diagnosis)'] was recorded prior to the specific survival time point in question, they were omitted from the pertinent survival analyses.
Replication	The source code for preprocessing and analyzing the data is available on GitHub (https://github.com/mertkarabacak/NCDB-G2G3_Glioma).
Randomization	N/A
Blinding	N/A

Reporting for specific materials, systems and methods

We require information from authors about some types of materials, experimental systems and methods used in many studies. Here, indicate whether each material, system or method listed is relevant to your study. If you are not sure if a list item applies to your research, read the appropriate section before selecting a response.

Materials & experimental systems

n/a	Involved in the study
☒	☐ Antibodies
☒	☐ Eukaryotic cell lines
☒	☐ Palaeontology and archaeology
☒	☐ Animals and other organisms
☐	☒ Clinical data
☒	☐ Dual use research of concern
☒	☐ Plants

Methods

n/a	Involved in the study
☒	☐ ChIP-seq
☒	☐ Flow cytometry
☒	☐ MRI-based neuroimaging

Clinical data

Policy information about clinical studies

All manuscripts should comply with the ICMJE guidelines for publication of clinical research and a completed CONSORT checklist must be included with all submissions.

Clinical trial registration	N/A
Study protocol	N/A
Data collection	The NCDB-Brain Participant User File (PUF) was filtered for adults at least 18 years of age diagnosed with histologically confirmed cranial WHO grade II and III gliomas between 2010 and 2017.
Outcomes	We built separate prediction models to predict patient survival outcomes for WHO grade II and grade III glioma patients at four distinct time points post-diagnosis: 12, 24, 36, and 60 months. The performances of the models were evaluated both visually and numerically. The visual assessment was completed using the ROC and PRC. The numerical evaluation involved metrics such as sensitivity, specificity, accuracy, area under the PRC (AUPRC), and AUROC. Furthermore, we assessed the calibration of our models utilizing the Brier score.
